# NaMYC2 transcription factor regulates a subset of plant defense responses in *Nicotiana attenuata*

**DOI:** 10.1186/1471-2229-13-73

**Published:** 2013-05-01

**Authors:** Melkamu G Woldemariam, Son Truong Dinh, Youngjoo Oh, Emmanuel Gaquerel, Ian T Baldwin, Ivan Galis

**Affiliations:** 1 Department of Molecular Ecology, Max Planck Institute for Chemical Ecology, Hans Knöll Straße 8, D-07745, Jena, Germany; 2 Present address: Institute of Plant Science and Resources, Okayama University, 2-20-1, Kurashiki 710-0046, Japan

**Keywords:** bHLH, Manduca sexta, MYC2, Transcription factors, Nicotiana attenuata, Nicotine, Phenolamides, Plant-insect interactions, Transcriptional regulation

## Abstract

**Background:**

To survive herbivore attack, plants have evolved potent mechanisms of mechanical or chemical defense that are either constitutively present or inducible after herbivore attack. Due to the costs of defense deployment, plants often regulate their biosynthesis using various transcription factors (TFs). MYC2 regulators belong to the bHLH family of transcription factors that are involved in many aspects of plant defense and development. In this study, we identified a novel MYC2 TF from *N. attenuata* and characterized its regulatory function using a combination of molecular, analytic and ecological methods.

**Results:**

The transcript and targeted metabolite analyses demonstrated that NaMYC2 is mainly involved in the regulation of the biosynthesis of nicotine and phenolamides in *N. attenuata*. In addition, using broadly-targeted metabolite analysis, we identified a number of other metabolite features that were regulated by NaMYC2, which, after full annotation, are expected to broaden our understanding of plant defense regulation. Unlike previous reports, the biosynthesis of jasmonates and some JA-/NaCOI1-dependent metabolites (e.g. HGL-DTGs) were not strongly regulated by NaMYC2, suggesting the involvement of other independent regulators. No significant differences were observed in the performance of *M. sexta* on *MYC2*-silenced plants, consistent with the well-known ability of this specialist insect to tolerate nicotine.

**Conclusion:**

By regulating the biosynthesis of nicotine, NaMYC2 is likely to enhance plant resistance against non-adapted herbivores and contribute to plant fitness; however, multiple JA/NaCOI1-dependent mechanisms (perhaps involving other MYCs) that regulate separate defense responses are likely to exist in *N. attenuata*. The considerable variation observed amongst different plant families in the responses regulated by jasmonate signaling highlights the sophistication with which plants craft highly specific and fine-tuned responses against the herbivores that attack them.

## Background

In their natural habitats, plants are exposed to a number of abiotic (e.g. drought, ultra-violet radiation, salinity) and biotic (e.g. herbivore and/or pathogen attack, competition) stresses which strongly undermine their Darwinian fitness. To cope with herbivory, plants have evolved intricate defense mechanisms that include mechanical barriers, trichomes, thorns, latex, waxes, and a toxic-/anti-nutritive chemical arsenal deployed either constitutively (e.g. nicotine, glucosinolates) or following herbivore attack (e.g. hydroxygeranyllinalool-diterpene glycosides (HGL-DTGs), phenolamides, trypsin protease inhibitors) [1-3]. In addition, and in concert with these direct defenses, plants recruit predators or parasitoids of the attackers using informative volatile organic compounds or nutritional rewards [4-6]. However, the costs of defense responses [2,7,8] necessitate the development of stringent regulatory mechanisms and several families of plant transcription factors (TFs) (e.g. ERF, bZIP, MYB, bHLH and WRKY) have been shown to regulate plant defense against biotic and abiotic stresses [9-11]. Many of these transcription factors are co-induced in response to different stresses suggesting the existence of complex interaction [12-14].

In many plant species, the role of phytohormones in coordinating the development of defense responses has clearly been shown, frequently with cross-talk among them to achieve intricately fine-tuned response outcomes [15-18]. Specifically, the jasmonate signaling pathway plays a critical role in mediating defense responses against herbivores [19-21]. In response to herbivore attack, GLA1 enzymes release 18:3 α-linolenic acid (α-LeA) from chloroplast membranes. α-LeA is subsequently converted to oxophytodienoic acid (OPDA) in the chloroplasts by lipoxygenase (LOX), allene oxide synthase (AOS) and allene oxide cyclase (AOC) enzymes. OPDA is transported to peroxisomes and oxidized by OPDA reductase (OPR) forming jasmonic acid (JA). In the cytosol, JA is conjugated to isoleucine by JAR enzymes that produce the bioactive jasmonate, (+)-*7-iso*-jasmonoyl-L-isoleucine (JA-Ile) [22,23]. JA-Ile associates with the SCF^COI1^ complex, presumably to ubiquinate JAZ repressors and tag them for degradation by the 26S proteasome. In the absence of stressful conditions, MYC2 is repressed by the JAZ repressors, which recruit TOPLESS (TPL) as a co-repressor either directly through the EAR (Ethylene Response Factor-Associated Amphifilic Repression) motif or using the EAR motif of the NINJA (Novel Interactor of JAZ) protein [24,25]. Degradation of JAZ proteins releases the MYC2 transcription factor from repression and reconfigures downstream transcriptional processes [11,24,26-28].

MYC2 is a member of the basic Helix-Loop-Helix (bHLH) family of transcription factors (TFs) [29,30] that are characterized by structurally and functionally conserved domains in many plant species. One of these conserved domains, the basic (b) region, is used to bind to variants of the G-box hexamer (5'-CACNTG-3') found on the promoters of MYC2-regulated genes. The HLH and ZIP domains are used for homo-/hetero-dimerization, while the JID (JAZ Interacting Domain) domain is used to interact with JAZ proteins [11,28,29,31-34].

MYC2 transcription factors participate in the regulation of many JA-dependent physiological processes: defense against herbivores/pathogens, drought tolerance, circadian clock, light signaling and root growth [11,35-39]. Guo et al. [40], in a proteomic study that involved mock- or MeJA-treated wild type and myc2 plants, recently identified 27 differentially regulated, JA-inducible and MYC2 dependent proteins involved in glucosinolate metabolism (22%), stress and defense (33%), photosynthesis (22.2%), carbohydrate metabolism (7.4%), protein folding and degradation (11.1%), highlighting the very diverse roles of MYC2.

*N. attenuata* is a wild tobacco species native to the Great Basin Desert in Utah (USA) which our group has developed into an ecological plant model. The defense responses of this species against its specialist herbivore, *Manduca sexta,* are well studied, and include the production of potent secondary metabolites: nicotine, HGL-DTGs, phenolamides and protease inhibitors [10,41-47]. In this study, we identified a putative MYC2 transcription factor in *N. attenuata* (NaMYC2) and characterized its role in defense response regulation using reverse genetic, transcriptomic and untargeted/targeted metabolomic approaches. Our transcriptomic and metabolomic data indicate a strong involvement of NaMYC2 in nicotine accumulation. However, silencing this gene had only a limited effect on the accumulation of other plant defense metabolites which strongly implicates the involvement of multiple independent and/or redundant transcriptional regulators in defense signaling of *N. attenuata* plants.

## Results and discussion

### NaMYC2 *transcripts are induced after herbivory*

**Figure 1:**
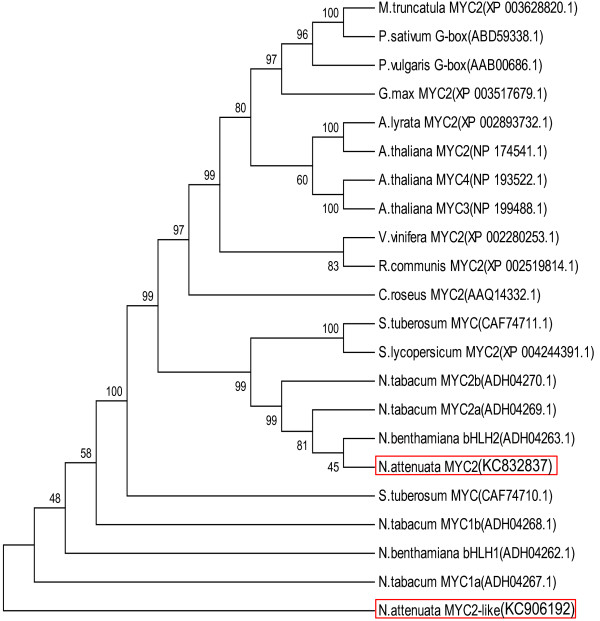
**Phylogeny of MYC2 transcription factors.** Protein sequences with high similarity to the *N. attenuata* MYC2 were retrieved from NCBI by Blast. Sequence alignment and phylogeny reconstruction were performed on MEGA5 using CLUSTAL W and Maximum Likelihood packages, respectively. The consensus tree generated was tested by bootstrapping (1000 times).

**Figure 2:**
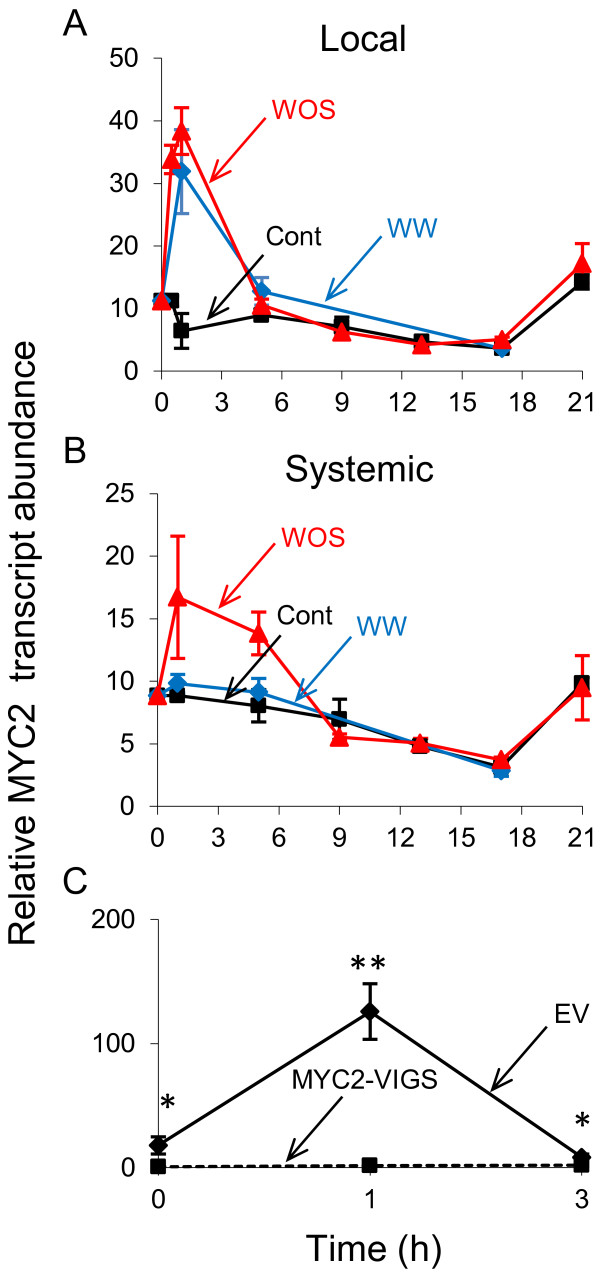
**Transcript abundance and silencing efficiency of MYC2 transcription factor in *N. attenuata.*** Rosette stage leaves (*n*=3) of wild type *N. attenuata* plants were treated with WW (blue line) or WOS (red line) or left untreated (black) and transcript abundances (mean ± SE) of MYC2 TF were measured by microarrays in (**A**) treated and (**B**) untreated systemic leaves (data were extracted from a previously published microarray dataset by Kim et al. [53]). (**C**) Using Virus Induced Gene Silencing (VIGS), the accumulation of *MYC2* transcripts were knocked down and the efficiency of silencing was determined by measuring the relative *MYC2* transcript abundances (mean ± SE; *n*=5) in control and WOS-induced EV (solid line) and MYC2-VIGS (dashed) plants by qRT-PCR. Asterisks indicate statistically significant differences (ANOVA, P < 0.05).

Herbivore attack induces a transient reconfiguration of plants' transcriptome, which translates into a reconfiguration of the metabolome. In *N. attenuata*, transcripts of genes involved in defense against herbivores are induced after both WW and WOS treatments. Interestingly, many transcripts show stronger responses to WOS, especially in systemically induced tissues [48-51]. In previous studies, the function of MYC2 TFs (Figure 1) in plant defense regulation was demonstrated; however, the detailed regulatory mechanisms differ amongst different plant species [11,31,37,52]. In wild type *N. attenuata* plants, transcripts of *MYC2* (GenBank Accession number KC832837) were transiently up-regulated in treated local leaves after both wounding (WW) and simulated herbivory (WOS). In contrast, in untreated systemic leaves, *MYC2* transcripts were up-regulated only after WOS treatment (Figure 2A and B), consistent with the differential response of herbivory-regulated genes to WW and WOS. These findings strongly suggested the involvement of NaMYC2 TF in plant defense against herbivores in *N. attenuata*[53]. Hence, to determine the function(s) of MYC2 in *N. attenuata*, we used a reverse genetic approach to knock down the accumulation of *NaMYC2* transcripts (by Virus Induced Gene Silencing, VIGS) and characterized the inoculated plants after verifying the efficiency of the VIGS procedure. Compared to empty vector (EV; transformation control) plants, a significant reduction was observed in *NaMYC2* transcript accumulation in MYC2-VIGS plants before (ANOVA, F_1,6_=339.22, *P*=0.0001) or 1 h (ANOVA, F_1,8_=418.72, *P*=0.0001) or 3 h (ANOVA, F_1,3_=42.41, *P*=0.007) after WOS induction (Figure 2C). As we also identified another MYC2 transcription factor (putatively named as MYC2-like; GenBank Accession number KC906192) with a considerable protein sequence similarity to MYC2, we tested if its transcript accumulation was affected in MYC2-VIGS plants. As expected from the positioning of the MYC2 silencing region in non-translated 3’ UTR of the gene, we found no significant reduction in the accumulation of the *MYC2-like* transcripts in MYC2-VIGS plants compared to EV control plants, indicating that VIGS silencing was confined to MYC2 TF (Additional file 1: Figures S1 and S2). In subsequent experiments, we used the silenced plants to determine the regulatory roles of MYC2 in plant defense in *N. attenuata*.

### Targeted analysis of secondary metabolite accumulation in MYC2-VIGS plants

Nicotine, phenolamides, hydroxygeranyllinalool diterpene glycosides (HGL-DTGs) and phenolic compounds are among the potent, JA-dependent anti-herbivore compounds in *N. attenuata*[2,10,54,55]. Their JA-dependent pattern of accumulation suggests that the biosynthesis of these compounds might be regulated by NaMYC2. To test this hypothesis, we used the MYC2-VIGS plants: previously, Saedler and Badwin [56] demonstrated that VIGS effectively knocks-down the expression of plant defense genes (e.g. *PMT*) in both leaves and roots of *N. attenuata* plants. Then, we used a targeted metabolomic approach to compare the accumulation of defensive secondary metabolites in untreated control and WOS-treated (24, 48 and 72 h) EV and MYC2-VIGS plants.

### Nicotine

**Figure 3:**
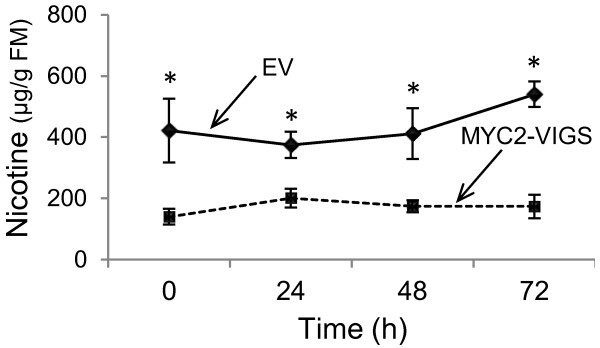
**Accumulation of nicotine in EV and MYC2-VIGS plants.** Metabolites were extracted from leaves (*n*=5) of EV- and MYC2-VIGS plants which were collected before or 24 h, 48 h or 72 h after WOS treatment and the average (mean ± SE) accumulation of nicotine was analyzed by HPLC-PDA. Asterisks indicate significant statistical differences (ANOVA, P < 0.05).

Nicotine is one of the most prominent chemical defense compounds in *N. attenuata*[57] and most of the genes involved in its biosynthesis have already been identified [52,57]. Nicotine is synthesized in roots and transported to leaves. To test if MYC2 regulates herbivore-induced biosynthesis of nicotine in *N. attenuata*, we measured the accumulation of nicotine in untreated or WOS-treated EV and MYC2-VIGS plants on HPLC-PDA. We found that compared to EV plants, the accumulation of nicotine was significantly lower before (ANOVA, F_1,7_=6.94, *P*=0.03) or 24 h (ANOVA, F_1,7_=10.06, *P*=0.01), 48 h (ANOVA, F_1,8_=17.53, *P*=0.003) and 72 h (ANOVA, F_1,8_=28.81, *P*=0.0007) after WOS treatment in MYC2-VIGS plants (Figure 3). Similar results were observed in another independent VIGS experiment (Additional file 1: Figure S3A, B) demonstrating that nicotine biosynthesis is strongly regulated by the MYC2 TF in *N. attenuata*. In addition to nicotine, we found MYC2-specific differences in the accumulations of two other alkaloids, anatabine and cotinine, as determined by a more selective and sensitive LC-TOF/MS method (Additional file 1: Figure S3C, D). Interestingly, while the ion intensities of anatabine and nicotine reduced in MYC2-VIGS leaves, cotinine accumulation increased.

Overall, our results are consistent with the previous reports which demonstrated regulation of jasmonate-induced nicotine/alkaloid biosynthesis by MYC2 TFs. In *N. tabacum* Bright Yellow (BY-2) cells that were transformed with an inverted-repeat (ir)NtMYC2a/2b construct, the accumulations of nicotine and anatabine were significantly reduced compared to untransformed controls [58]. The NtMYC2 protein was also shown to regulate nicotine biosynthesis either by directly binding to the promoters of nicotine biosynthetic genes in roots or activating NtERF189 which, in turn, activates genes involved in nicotine biosynthesis [52]. In *N. benthamiana*, VIGS of two bHLH transcription factors (named NbbHLH1 and NbbHLH2) as well as NbERF1 and NbHB1 decreased MeJA-induced accumulation of nicotine [59]. These results demonstrate both the regulatory functions of MYC2 and the involvement of a network of transcription factors in the regulation of nicotine biosynthesis. However, the functions of the tobacco *MYC2* genes were not examined in the context of natural herbivore feeding; neither were the effects of these *MYC2* genes on the accumulations of other tobacco defense metabolites (e.g. phenolamides, HGL-DTGs, etc.) studied. From the phylogenetic relationship of MYC/bHLH TFs in *N. attenuata, N. tabacum* and *N. benthamiana* (Figure 1) and our results, the presence of additional MYC TFs in *N. attenuata* is a reasonable prediction. Further characterization of these putative TFs might help to fully understand the biosynthesis and ecological consequences of nicotine/alkaloid biosynthesis. Moreover, characterization of additional regulators would complement the partial regulatory function of NaMYC2 in the control of different classes of *N. attenuata* defense metabolites, as demonstrated in the next sections.

### Phenolamides

**Figure 4:**
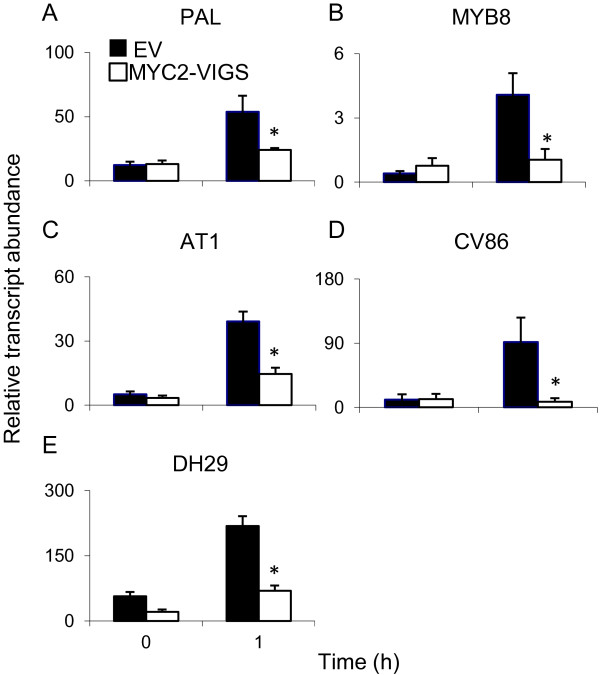
**Transcript accumulation of selected genes involved in phenolamide biosynthesis in EV and MYC2-VIGS *N. attenuata *plants.** Transcript abundance (mean ± SE; *n*=5) of genes involved in JA-dependent phenolamide biosynthesis was determined in WOS-induced EV and MYC2-VIGS plants. Quantification was performed by qRT-PCR using the house-keeping gene, *Elongation Factor 1α* (*EF-1α*), for normalization. One h after WOS-treatment, significant reductions (ANOVA, P < 0.05, indicated by asterisks) were observed in transcript accumulation of *PAL* (**A**), *MYB8* (**B**), *AT1* (**C**), *CV86* (**D**) and *DH29* (**E**) in MYC2-VIGS plants.

Recently, regulation of the biosynthesis of phenolamides by NaMYB8 TF and its ecological relevance were reported in *N. attenuata*[10,47]. Considering a previous report in *A. thaliana* which indicated regulation of MYB TFs by AtMYC2 [11] and our microarray data which identified a MYB TF among the NaMYC2-regulated genes (Additional file 2: Table S1), we reasoned that, in *N. attenuata*, NaMYB8 or the genes it regulates might be regulated by NaMYC2. To test this possibility, we treated EV and MYC2-VIGS plants by WOS and measured the relative transcript abundances of NaMYB8 and downstream genes involved in phenolamide biosynthesis. The transcript accumulations of these genes did not differ between EV and MYC2-VIGS plants in untreated plants (0 h); however, 1 h after WOS treatment, a significant reduction was observed in transcript accumulations of *NaMYB8* (ANOVA, F_1,6_ = 9.81, *P* = 0.02), *NaPAL* (ANOVA, F_1,6_ = 17.14, *P* = 0.006), *NaAT1* (ANOVA, F_1,6_ = 16.00, *P* = 0.007), *NaDH29* (ANOVA, F_1,6_ = 28.25, *P* = 0.001) and *NaCV86* (ANOVA, F_1,6_ = 6.66, *P* = 0.04) in MYC2-VIGS plants (Figure 4). Our data and the previously demonstrated regulation of *NaAT1*, *NaDH29*, *NaCV86* by *NaMYB8*[47] point to the possibility that NaMYC2 controls phenolamide biosynthesis by regulating the expression of *NaMYB8*.

**Figure 5:**
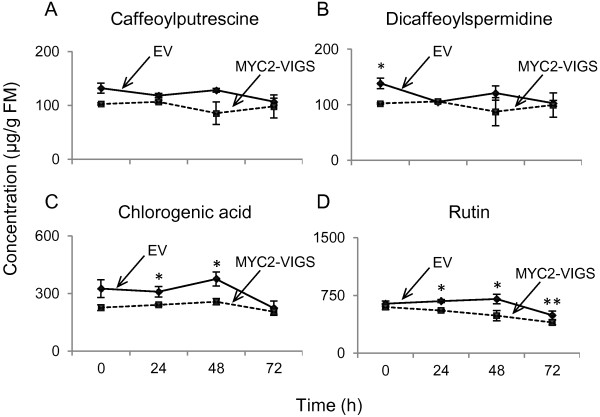
**Targeted analysis of phenolamide accumulation in EV and MYC2-VIGS plants.** Following the extraction of metabolites from leaves (*n*=5) of EV- and MYC2-VIGS plants that were collected before or 24 h, 48 h or 72 h after WOS treatment, the average (mean ± SE) accumulations of caffeoylputrescine (**A**), dicaffeoylspermidine (**B**), chlorogenic acid (**C**) and rutin (**D**) were analyzed by HPLC-PDA. Asterisks indicate significant (ANOVA, P < 0.05) statistical differences.

Next, we measured the WOS-induced accumulation of caffeoylputrescine, dicaffeoylspermidine, chlorogenic acid and rutin in EV and MYC2-VIGS plants to test if the accumulation of these compounds followed the observed NaMYC2-dependent transcript accumulation patterns. Surprisingly, we found very few significant differences between EV and MYC2-VIGS samples (Figure 5), which was also confirmed in an independent VIGS experiment (Additional file 1: Figure S4A to D). In both VIGS experiments, due to time required for the efficient spread of silencing, the samples used to extract secondary metabolites from EV- and MYC2-VIGS plants were collected at the early flowering stage from positions corresponding to bleached parts on PDS-VIGS plants. Silencing of *phytoene desaturase* (PDS) leads to photo bleaching of leaves and allows for a visual verification of the spread of silencing that corresponds to clear white chlorophyll-less areas on the leaves. However, at late elongated/flowering stage, the inducible character of phenolamide accumulation is known to cease, although nothing is known about transcript accumulation at this stage [10]. Kaur et al., [10] showed that the highly inducible levels of caffeoylputrescine (a phenylpropanoid-polyamine conjugate) in the vegetative tissues of rosette and early-elongated stages of *N. attenuata* plants clearly shifted to the reproductive tissues after flowering and capsule development. Consequently, hardly any caffeoylputrescine was detected in the leaves of mature plants. Thus, due to the "constitutive" and localized nature of phenolamide accumulation in later stages of plant development, their biosynthesis may not be strongly influenced by NaMYC2. Alternatively, even though the transcription of the biosynthetic enzymes remains inducible at later stages of development (Figure 4), translation/post-translational modifications of the enzymes might not occur or the necessary substrates, such as phenylpropanoids and polyamines, could be diverted to other important functions in flowering plants. Finally, it is possible that our ability to detect MYC2-dependent differences was masked because of the plants' response to the VIGS process (i.e. virus infection that may induce phenolamide biosynthesis) or that the level of silencing was not sufficient to affect phenolamide biosynthesis. The disconnect between transcript and metabolite data could be, therefore, explained by the dynamic and/or synergistic regulation of phenolamide biosynthesis; developmentally and by herbivore/pathogen attack. We hypothesized that NaMYC2 is likely to be involved in the regulation of phenolamide biosynthesis in younger plants. However, this could not be tested in the current experimental setup (using MYC2-VIGS plants) and will require the generation of stably transformed plants.

### Total hydroxygeranyllinalool diterpene glycosides (HGL-DTGs) and TPI levels

**Figure 6:**
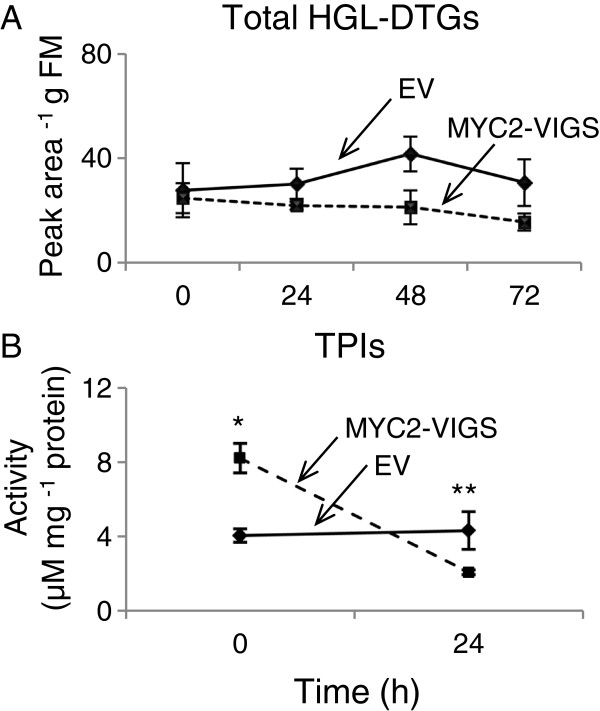
**WOS-induced accumulation of HGL-DTGs and TPI activity in EV and MYC2-VIGS plants.** Leaves (*n*=5) of EV- and MYC2-VIGS plants were treated with WOS for 24 h, 48 h or 72 h or left untreated (0 h) and collected to extract and analyze accumulation (mean ± SE) of total HGL-DTGs (**A**) on HPLC-PDA. Using un-induced (0 h) and WOS-induced (24 h) samples from the same experiment, TPI activity (**B**) was determined using a radial diffusion assay. Asterisks indicate significant statistical differences.

HGL-DTGs are JA-dependent metabolites with well-demonstrated roles in plant defense against herbivores in *N. attenuata*[55,60-62]. To determine if herbivore-induced accumulation of HGL-DTGs was regulated by MYC2 in *N. attenuata*, we treated EV and MYC2-VIGS plants with WOS, extracted metabolites and analyzed total HGL-DTGs by HPLC-PDA. We found no significant difference in the accumulation of total HGL-DTGs in control plants or plants treated with WOS for 24, 48 or 72 h (Figure 6A and Additional file 1: Figure S4E), indicating that MYC2 may not be involved in regulating the biosynthesis of this class of compounds. We used a radial diffusion assay [63] to compare the WOS-induced TPI activity between EV and MYC2-VIGS plants and found that, although TPI activity levels were significantly reduced 24 h after WOS treatment, the levels were higher in MYC2-VIGS plants prior to induction; and this did not correlate with *MYC2* expression (Figure 6B).

Taken together and considering the JA-/COI1-dependence of HGL-DTG and TPI accumulation in *N. attenuata*[64], the biosynthesis of HGL-DTGs and TPIs in *N. attenuata* is likely regulated by a JA-dependent, but NaMYC2-independent mechanism. Alternatively, the function and/or synergism of an independent *MYC2* gene in *N. attenuata* can explain the partial function of NaMYC2. In addition, similar to phenolamides, the accumulation of HGL-DTG and TPI is also strongly influenced by the developmental stage of the plants [65]. Van Dam et al. [66] showed that the *de novo* synthesis of PIs is limited to the early stages of plant development and that flowering plants treated with methyl jasmonate did not significantly increase their local or systemic PI activity levels. In addition, damage to older leaves elicited a much weaker systemic response in younger leaves compared to younger source leaves, a pattern also reported from other studies in *N. tabacum*[67]. Heiling et al. (2010) demonstrated that the concentrations of 17-hydroxygeranyllinalool diterpene glycosides (DTGs) were highest in most valuable young and reproductive tissues, which is required for effective defense of these tissues against herbivores in *N. attenuata*.

### NaMYC2 and regulation of herbivory-induced phytohormone accumulation

**Figure 7:**
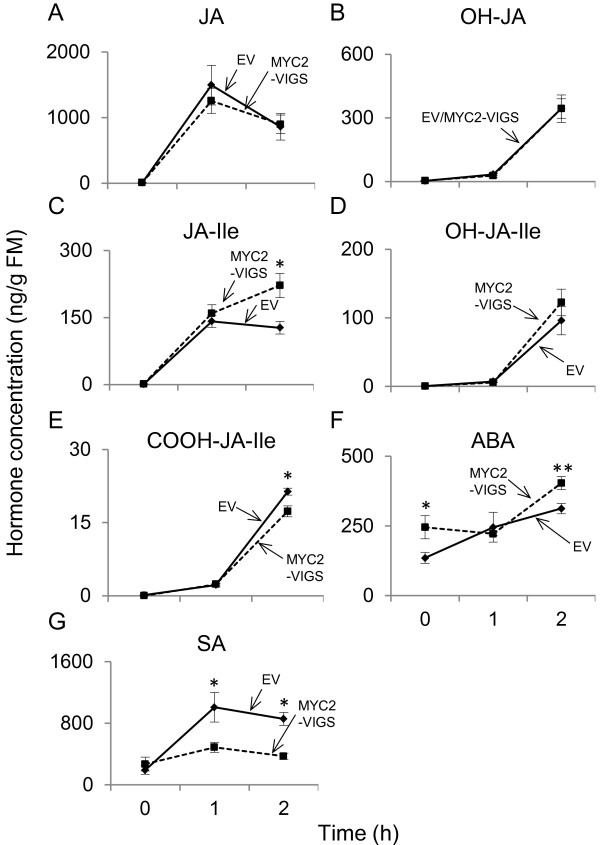
**Herbivore-induced accumulation of phytohormones in EV and MYC2-VIGS plants.** Fully elongated leaves (*n*=5) of EV and MYC2-VIGS plants were collected before or 1 h or 2 h after WOS-treatment for phytohormone extraction. Extracts were analyzed on LC-MS^3^ and the levels (mean ± SE) of JA (**A**), OH-JA (**B**), JA-Ile (**C**), OH-JA-Ile (**D**), COOH-JA-Ile (**E**), ABA (**F**) and SA (**G**) were determined. Different letters indicate statistically significant differences.

In *A. thaliana*, MYC2 regulates genes involved in the biosynthesis of phytohormones and contributes to the feedback loop in jasmonate biosynthesis. MYC2 also regulates its own transcription, presumably to further enhance jasmonate responses [11,38]. Hence, we asked if NaMYC2 contributed to the biosynthesis or metabolism of phytohormones in *N. attenuata*, and to address this question, we measured the accumulation of jasmonates in untreated and WOS-treated EV and MYC2-VIGS plants in two independent VIGS experiments. In summary, no consistent, MYC2-dependent differences were observed in the accumulation of JA, OH-JA, JA-Ile, OH-JA-Ile and COOH-JA-Ile among EV and MYC2-VIGS plants; neither did we detect consistent differences in the accumulations of ABA or SA (Figure 7, Additional file 1: Figure S5). In agreement with these observations and unlike in *A. thaliana*[11], we did not find significant changes in transcript accumulation of any of the genes involved in the biosynthesis/metabolism of these phytohormones in our microarray data (Additional file 2: Table S1). From these observations, we conclude that, in *N. attenuata*, MYC2 does not regulate the biosynthesis and/or metabolism of jasmonates, ABA or SA.

### Performance of the specialist herbivore on MYC2-VIGS plants

**Figure 8:**
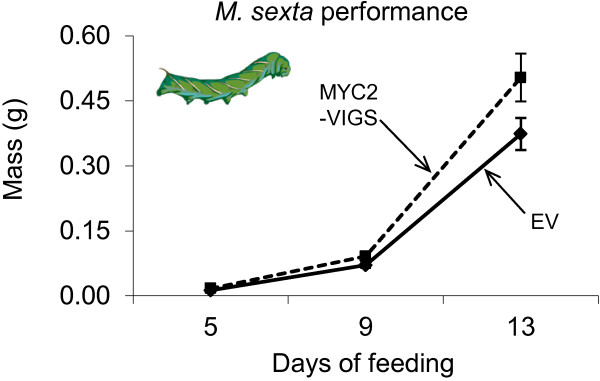
**Performance of *M. sexta *on EV- and MYC2-VIGS plants.** Neonates (*n*=20) of the specialist herbivore, *M. sexta,* were fed on EV and MYC2-VIGS *N. attenuata* plants for 13 d and their masses (mean ± SE) were determined on the 5^th^, 9^th^ and 13^th^ day to compare their relative growth performance. No significant differences were observed in mass gained among caterpillars fed on EV or MYC2-VIGS plants.

As a key regulator of plant defense responses, we asked if the performance of the specialist herbivore, *M. sexta* was affected by *MYC2* silencing. Consequently, we fed neonates (*n* = 20) of *M. sexta* on EV and MYC2-VIGS plants for 13 d measuring their masses every 4 d. At all measurement times, we observed no significant difference in the mass gained by caterpillars when fed on EV or MYC2-VIGS plants (Figure 8). This is consistent with the observation that in MYC2-VIGS plants, significant changes were observed only in the accumulation of nicotine, a metabolite to which neonates of *M. sexta* are very tolerant. In contrast, in a manner that was also consistent with the patterns of metabolite accumulation in irCOI1 plants, neonates of *M. sexta* fed on COI1-silenced plants gained significantly more mass compared to those fed on WT plants [64]. The question is, hence, whether there are other JA/COI1-in/dependent MYC2 (or other) TFs that regulate these other defense metabolites of *N. attenuata* that are particularly important for the performance of *M. sexta* larvae.

### Large scale transcriptomic and metabolomic analysis of MYC2-silenced leaves

The role of MYC2 TFs in orchestrating plant defense and developmental processes in several plant species were previously reviewed [35,68,69]. As master regulators, MYC2 TFs may either directly regulate the genes responsible for defense metabolite biosynthesis or regulate their regulators [11,68]. To provide information for further work, we used unbiased approaches and compared herbivore-induced (WOS) changes in the transcriptome and metabolome of EV and MYC2-VIGS *N. attenuata* plants.

### *NaMYC2 regulated transcriptome of* N. attenuata

**Figure 9:**
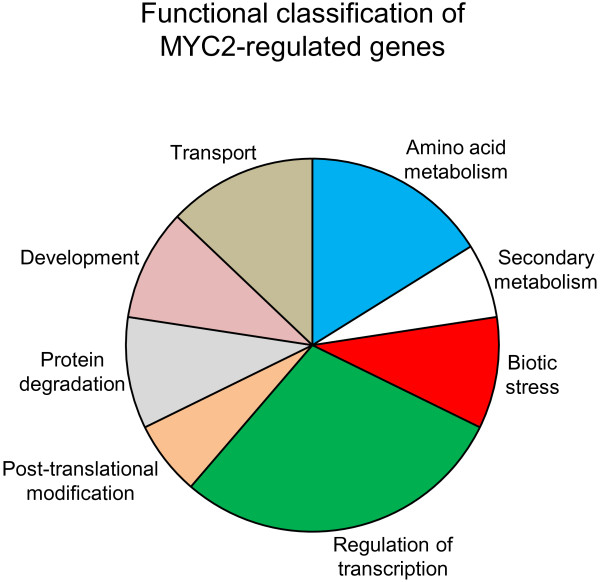
**Transcriptional regulation by MYC2 transcription factor.** Rosette leaves (*n*=3) were collected from WOS-induced (1 h) EV and MYC2-VIGS *N. attenuata* plants for microarray analysis. After pre-processing the raw data, genes whose expression changed significantly among the genotypes were identified using Significance Analysis of Microarrays (SAM) package and functional annotation was performed on Blast2Go. Pie chart depicts the functional categories of MYC2-regulated genes in *N. attenuata*.

For transcriptomic analysis, we treated EV and MYC2-VIGS plants with WOS for 1 h and compared their respective induced transcriptome using microarrays. This approach, although unable to discover late induced metabolic genes could reveal the intermediate regulators and TFs downstream of NaMYC2. We normalized and log_2_-transformed the raw data, identified genes whose expressions were significantly altered in MYC2-VIGS plants (using Significance Analysis of Microarrays (SAM) package) and annotated them by Blast2Go. Compared to EV plants, the expressions of 47 genes were significantly (fold change of 2 or more) altered in MYC2-VIGS plants (Additional file 2: Table S1). When we grouped the regulated genes according to TAIR (The Arabidopsis Information Resource) functional annotation scheme, the genes were found to be involved in diverse physiological processes: regulation of transcription (20.45%), amino acid metabolism (11.3%), secondary metabolism (4.5%), biotic stress (6.8%), development (6.8%), transport (9.1%), post-translational modification (4.5%) and protein degradation (6.8%) (Figure 9, Additional file 2: Table S1). Specifically, several key regulators of plant defense responses, transcription factors (WRKY, MYB) or signaling components (calmodulin or calcium binding proteins) were among those identified by the microarray analysis. Close inspection of MYC2-regulated genes in *N. attenuata* identified additional early induced genes involved in defense against herbivores (terpene synthases and proteinase inhibitors) or pathogens (PR proteins) (Additional file 2: Table S1). Our data support the *A. thaliana* report in which the regulatory role of AtMYC2 on a spectrum of physiological processes was shown: from herbivore/pathogen defense to hormone biosynthesis; from primary and/or secondary metabolism [11,70] to photomorphogenic development [32,71].

In contrast to independently performed qRT-PCR measurement of transcript abundances of phenolamide biosynthetic genes, the microarray analysis did not identify these genes (*PAL*, *AT1*, *DH29* and *MYB8*) as differentially regulated in MYC2-VIGS plants compared to EV-VIGS plants because these genes did not pass the strict statistical criteria set for selection of at least 2-fold down-regulated genes in microarray experiment. Nicotine biosynthesis genes are only expressed in the roots and therefore could not be evaluated in the leaf samples used for microarrays.

### *Silencing of* NaMYC2 *significantly affects the* N. attenuata *metabolome*

**Figure 10:**
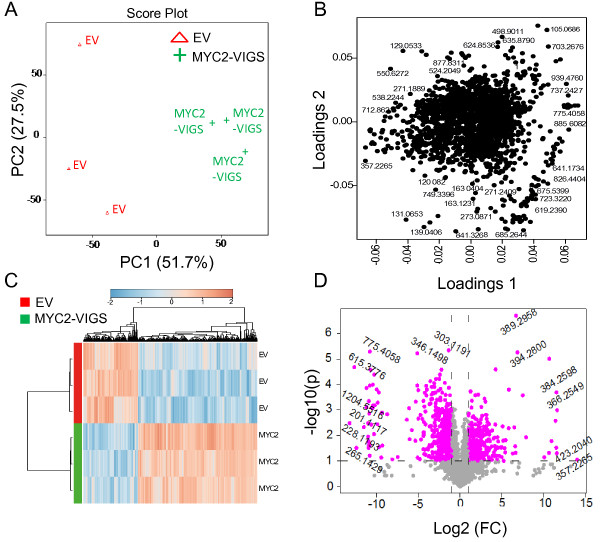
**Broadly targeted metabolomic analysis of herbivore-induced EV and MYC2-VIGS plants.** Leaves (*n*=3) were collected from caterpillar-attacked (4 d) EV and MYC2-VIGS plants and used for untargeted metabolomic analysis with an HPLC-TOF-MS. Raw data were pre-processed by XCMS and CAMERA packages and a PCA plot (**A**) was generated based on the molecular features that differed significantly (fold change > 2, P < 0.05) among the indicated genotypes. Principal component 1 (PC 1) explains 51.7% of the variance while PC 2 explains 27.5%. The contribution of the molecular features to the PCA clusters is shown by the loading plot (**B**) while the volcano plot (**D**) depicts important features with a fold change and *t*-test threshold of 2 and 0.1, respectively. (**C**) A heat map depicts the expression of regulated molecular features in EV and MYC2-silenced plants.

Do MYC2-mediated changes in the herbivore-induced transcriptome translate into a wider spectrum of defense secondary metabolites, apart from alkaloids already demonstrated by targeted analytical approach? We used an unbiased metabolomic profiling approach by HPLC/ESI-TOF-MS and analyzed metabolites extracted from leaves of EV and MYC2-VIGS plants that were continuously attacked (4 d) by neonates of *M. sexta*. The raw data were normalized, log_2_-transformed and preprocessed using XCMS and CAMERA packages as described in the Methods section. To visualize the direction of the total variability in our samples without taking the class labels into consideration, we used an unsupervised approach (Principal Component Analysis, PCA) and observed that EV and MYC2-VIGS samples were separated to two clusters by PCA, suggesting genotype-specific differences at the level of metabolites (Figure 10A). The features that contributed strongly to PC1 (which explains 51.7% of the total variability) and PC2 (which explains 27.5% of the total variability) are depicted in the loading plot (Figure 10B). When we screened for metabolic features that differed among the genotypes (fold changes of 2 or more), we identified 897 features; 741 of which differed significantly (*t*-test threshold of 0.05 or less) between EV and MYC2-VIGS plants (Additional file 3: Table S2). The overall pattern of regulation can be visualized from the heat map (Figure 10C) generated on Metaboanalyst 2.0 using the significant metabolic features (Ward clustering algorithm and Pearson distance measures). In total, 712 metabolite features that met both fold change and *t*-test thresholds (2-fold or more, P < 0.05, respectively) were identified and the most important features were plotted on the volcano plot (indicated by the purple dots) (Figure 10D, Additional file 3: Table S2). Some of these features (m/z 163.123, 132.082, 163.039) were previously annotated as molecular fragments of metabolites involved in plant defense against herbivores in *N. attenuata*[47,72]. However, identification and annotation of the remaining features remain as significant challenge for future experiments. Overall, our metabolomic analysis demonstrates the importance of MYC2 in the regulation of plant's metabolome and when these metabolomic features are annotated, it will be possible to precisely map the regulatory role of MYC2 on plant defense and developmental responses.

## Conclusions

In many plant species, attack from herbivores elicits a cascade of complex transcriptional and metabolic responses that improve plant defense. The effectiveness of plant defense depends on the efficiency by which the timing and duration of responses are regulated. In this study, we identified a MYC2 TF in *N. attenuata* and characterized its regulatory role using transcriptomic and metabolomic approaches. Transcriptionally, we showed that the expressions of many genes, including transcription factors, involved in plant development or defense responses were affected when MYC2 was silenced in *N. attenuata*. This was supported by the metabolomic data which identified a large number of differentially regulated molecular features following the silencing. Most importantly, as was previously reported in *N. tabacum* and *N. benthamiana*, we showed that NaMYC2 regulates the *in planta* accumulation of nicotine in *N. attenuata* leaves. The fact that MYC2 did not strongly affect the accumulation of other JA-dependent metabolites, HGL-DTGs and proteinase inhibitors, suggests that another MYC TF is likely involved in the process.

Despite the considerable conservation of the basic components of plant defense responses among different plant species, substantial variations exist in the responses outcomes which highlights between-species differences in downstream regulatory fine-tuning [31,73]. For example, in contrast to the considerable similarity among members of the genus *Nicotiana* in the regulation of nicotine biosynthesis by MYC2 [52,58,59] (Figure 1), silencing MYC2 in *N. attenuata* did not have the exact same effects as reported in *A. thaliana*; we did not observe a role of MYC2 either in a positive feedback loop activating JA biosynthesis or in a negative feedback involving suppression of the jasmonate response through the activation of JAZ repressors [11,74].

In addition, not all JA-dependent defense metabolites (e.g. HGL-DTGs) were regulated by MYC2 in *N. attenuata*. In fact, when compared against the diversity of defense metabolites in *N. attenuata*, the regulatory function of MYC2 is quite limited. This rather limited role suggests that other members of the bHLH family of transcription factors might be involved in the regulation of defense responses not regulated by MYC2. The recent identification of additional MYC2 TFs in *A. thaliana*[36,37], *N. tabacum*[52,58] and *N. benthamiana*[59] with overlapping or distinct functions support this conjecture.

Indeed, we found an additional MYC2-like gene (KC906192) in diploid *N. attenuata* showing a 72.3% protein sequence identity with NaMYC2. In the phylogenetic analysis, MYC2-like protein clustered separately from the MYC2 clade of Solanaceae species, including *N. tabacum* MYC2a and MYC2b. When we briefly examined the function of *MYC2-like* gene in *N. attenuata*, interestingly, increased defense responses in MYC2-like-VIGS plants were observed (data not shown). This was in a strong contrast to silencing the NaMYC2 (and *N. benthamiana* genes *bHLH1*and *bHLH2*; [59]) but in agreement with the VIGS–induced silencing of the *N. benthamiana* bHLH3 (a gene fragment not included in phylogenetic tree shown in Figure 1), which increased the nicotine content in the VIGS-silenced *N. benthamiana* plants after foliar application of MeJA [59]. Therefore, some of the MYC2-like genes may work as repressors of JA-induced responses, contributing to a fine-tuning of defense against herbivores, possibly by competing for promoter binding sites with the activator-type MYC2 genes. As previously demonstrated for the transient character of JA-Ile accumulation [62,75], tight control of JA signaling is likely to be essential for plant responses to multiple biotic stresses in the environment. Identification and characterization of additional MYC2 TFs in *N. attenuata* and other plant species is likely to provide a more complete mechanistic picture of JA-regulated defense responses.

Considering the high degree of conservation in the binding site of MYC2 TFs in different species [29,31], we believe future research in determining the binding sites of these TFs will be critical to understanding their function. When these binding sites are identified, additional MYC2-dependent genes or other transcription factors that respond to herbivory, disease, environmental stress or development can be more readily identified. It would be interesting to identify the interacting partners of MYC2 TFs in *N*. *attenuata* and characterize the mechanisms of interaction to understand how the signaling components evolved. In *A. thaliana*, transcriptional regulation by MYC2 requires interactions with important regulatory elements including members of the mediator complex proteins (e.g. MED25), chromatin-opening proteins like General Control Non-repressible 5 (GCN5), members of the histone acetyl transferase family and SPLAYED (SYD) [35,76,77]. Identification and characterization of homologues of these components in *N. attenuata* might test the generality of the signaling processes across different plant families.

## Methods

### Plant growth and treatments

*N. attenuata* seeds that were collected from its native habitat in Great Basin desert, Utah (USA) and inbred for 31 generations were used for the experiments. Seed germination and plant growth conditions were described in Krügel et al. [78]. To experimentally simulate herbivory, we wounded fully expanded leaves of EV and MYC2-VIGS (*n*=5) *N. attenuata* plants with a serrated fabric pattern wheel and the wounds were treated with 20 μL of diluted (1:5, v/v in water) *M. sexta* oral secretions (WOS), while controls were collected from untreated plants. To evaluate performance of the specialist herbivore (*M. sexta*) on transformed plants, freshly hatched neonates were fed on EV and transformed plants (*n* = 20) and their masses were measured every 4 d.

### Virus Induced Gene Silencing (VIGS)

Virus Induced Gene Silencing (VIGS) system, described in Saedler and Baldwin [56], was used to transiently silence MYC2 transcription factor. Briefly, we amplified ~250 bp fragment of the *N. attenuata MYC2* using specific primers (Additional file 4: Table S3), cloned them into the P^TV00^ vector. We verified the clone by sequencing and transformed GV3101 strain of *Agrobacterium tumefaciens* with either untransformed plasmid (P^TV00^, control) or plasmids harboring the inserts (p^TV-MYC2^) and incubated them at 26°C for two days. On the day of infiltration, overnight cultures of all constructs and p^BINTRA^ and p^TVPDS^ were inoculated into YEP media containing antibiotics (Kanamycin 50 mg/L) and incubated (28°C) for 5 h. When the cultures attained an OD of 0.6 to 0.8, we centrifuged them (1,125*g*, 4°C for 5 min), resuspended the pellets in an equimolar mix (5 mM) of MgCl_2_ and MES and prepared a 1:1 mix of each construct with the helper strain p^BINTRA^. Using 1 mL syringes, we infiltrated the suspension into five leaves of 25 d old *N. attenuata* plants, covered them with plastic and left them in a dark chamber for 2 d. The plants were kept in the growth chamber under 16 h/day, 8 h/night light regime at 22°C. We monitored the spread of silencing using control plants infiltrated with the p^TVPDS^ construct which induced leaf bleaching, while the efficiency of silencing was determined by measuring transcript abundances using qRT-PCR.

### Microarray analysis

We treated fully elongated leaves of EV and MYC2-VIGS plants (*n* = 3) with WOS for 1h, collected and ground the leaves in liquid nitrogen and extracted RNA for the microarray analysis as described in Gillardoni et al. [79]. After hybridization and array processing, we normalized (with the 75^th^ percentile of the respective columns) and log_2_-transformed the raw expression values obtained from the "gProcessedSignal" column and processed them using Significance of Microarrays (SAM; http://www-stat.stanford.edu/~tibs/SAM/) package on Excel (Microsoft). For the analysis, we set the minimum fold change, delta and median FDR (%) values to 2, 0.69 and 15.8 (%) respectively. Genes that differed significantly in comparison to EV plants were annotated using Blast2Go [80] and grouped according to TAIR classification. The microarray data was deposited in GEO under the accession number GSE45608.

### Transcript abundance measurement

We extracted total RNA from frozen leaf material of untreated or WOS-treated EV and MYC2-VIGS plants (*n* = 5) using TRIzol reagent (Invitrogen) as recommended by the manufacturer. We treated the total RNA with DNAse (RQ1 RNase-Free DNase; Promega) before synthesizing cDNA using oligo (dT)_18_ and Superscript II reverse transcriptase (Invitrogen). Transcript abundances were measured on Mx3005P Multiplex qPCR (Stratagene) with qPCR core kit for SYBR Green I (Eurogentec). Relative transcript abundances were determined by comparing sample fluorescence signals to dilution series of cDNA prepared from the 1 h WOS -treated samples, and examined on the same plate. Signals were then normalized by the average EF-1α transcript abundances determined separately for each sample. The primers used for qRT-PCR are listed in Additional file 4: Table S3. As there is a considerable similarity in protein coding sequences in multiple members in bHLH TF family, it may imply significant functional redundancy of these regulators in biological systems. We therefore carefully designed our primers in the 3’ non-translated end of the gene to amplify and detect specifically the NaMYC2 transcription factor (Additional file 1: Figure S2).

### Phytohormone analyses

Fully-expanded leaves of EV and MYC2-VIGS plants (*n* = 5) were treated with WOS for 1 h or 2 h, collected and ground in liquid nitrogen and stored at −80°C until use. We homogenized about 200 mg powder in 1 mL ethyl acetate (containing 200 ng/mL D_2_-JA and 40 ng/mL D_6_-ABA, D_4_-SA and JA-^13^C_6_-Ile internal standards), centrifuged for 20 min (16,100*g*, 4°C) and transferred the supernatants into new tubes. After re-extracting the pellets with 0.5 mL ethyl acetate and combining the supernatants, we evaporated the ethyl acetate on a vacuum concentrator (Eppendorf) and resuspended the residue in 0.5 mL 70% methanol in water (v/v). Then, we centrifuged the re-suspended samples for 10 min (16,100 g, 4°C) and analyzed the supernatant (10 μL) on Varian 1200L Triple-Quadrupole-LC-MS (Varian) using a ProntoSIL® column (C18; 5 μm, 50 × 2 mm; Bischoff) attached to a precolumn (C18; 4 × 2 mm, Phenomenex). Detail measurement conditions are described in Woldemariam et al. [62].

### Secondary metabolite analysis

To undertake targeted defense secondary metabolite (nicotine, total 17-hydroxygeranyllinalool diterpene glycosides [HGL-DTGs], caffeoylputrescine, dicaffeoylspermidine, chlorogenic acid and rutin) analysis, we treated leaves of EV and MYC2-VIGS (*n* = 5) plants with WOS for 24, 48 or 72 h, collected and ground the samples in liquid nitrogen. Control samples were collected without treatment. About 100 mg powder was extracted and analyzed on HPLC equipped with a photodiode array detector as previously described in Onkokesung et al. [81].

### Untargeted metabolomic analysis

To undertake an unbiased metabolomic analysis, metabolites were extracted from leaves (*n* = 3) of EV and *MYC2* silenced *N. attenuata* plants fed on for 4 d by neonates of *M. sexta* and analyzed on an HPLC 1100 Series system (Agilent, Palo Alto, USA) coupled to a MicroToF mass spectrometer (Bruker Daltonik, Bremen, Germany). The optimized analytic procedures are described in Gaquerel et al. [72]. Briefly, peak picking, peak detection and RT corrections were performed by XCMS (and CAMERA) package using the following parameters: centWave method; ppm = 20; snthresh =10; peakwidth = between 5 and 18 s; minfrac=0.5; minsamp=1; bw=10; mzwid=0.01; sleep=0.001. To fill missing features, we used the FillPeaks function from XCMS. We exported the pre-processed data to Excel, filtered those features with RTs < 60 seconds and m/z < 80 and analyzed the processed data on Metaboanalyst 2.0 following the procedure described before [82].

### Statistical analysis

We used STATVIEW (version 5.0; SAS Institute, Cary, NC, USA) software to perform statistical analyses with alpha level of 0.05 for all statistical tests.

## Competing interests

The authors verify that there are no competing interests.

## Authors’ contributions

MGW (designed experiments; conducted experiments; analyzed data; wrote manuscript); STD (conducted experiments); YO (conducted experiments); EG (designed experiments; analyzed data); ITB (designed experiments; wrote manuscript; provided financial support); IG (designed experiments; wrote manuscript). All authors read and approved the final manuscript.

## Supplementary Material

Additional file 1: Figure S1 (A) Nucleotide sequences of *NaMYC2* and *NaMYC2-like* (*MYC2L*) genes. (B) The relative accumulation (mean ± SE) of *MYC2-like* transcripts (*MYC2L*, n=5) was determined before (0 h) and after WOS treatment (1 h, 2 h) in EV (solid line) and MYC2-VIGS (dashed) *N. attenuata* plants by qRT-PCR. **Figure S2**. Alignment of nucleotide sequences of the *N. attenuata* MYC2 and MYC2-like TFs. Sequences were aligned by EMBOSS Stretcher program (http://www.ebi.ac.uk/Tools/psa/) and regions used for silencing of *MYC2* and determination of transcript abundances were highlighted in color. **Figure S3**. (A) Control and WOS-treated leaves (*n* = 5) of EV and MYC2-VIGS plants were collected and used to analyze the accumulation (mean ± SE) of nicotine. (B) to (D): Leaves (*n =* 3) of EV and MYC2-VIGS plants attacked by neonates of *M. sexta* for 4 d were collected, extracted and analyzed for metabolites by HPLC-TOF-MS. The extracted ion chromatographs (EIC) for nicotine (B), anatabine (C) and cotinine (D) were overlaid to compare the regulation of alkaloid biosynthesis by MYC2. **Figure S4**. Secondary metabolite accumulations in EV and MYC2-VIGS plants before (0 h) or 24, 48 and 72 h after WOS treatment. Control and WOS-treated leaves (*n* = 3) of EV and MYC2-VIGS plants were collected and used to analyze the accumulation (mean ± SE) of caffeoylputrescine (A), dicaffeoylspermidine (B), chlorogenic acid (C), rutin (D) and total HGL-DTGs (E) on HPLC-PDA. **Figure S5**. Accumulation of phytohormones in EV and MYC2-VIGS plants. Fully elongated leaves of EV and MYC2-VIGS plants were treated with WOS and harvested after 1, 2, and 3 h, or collected without treatment. The accumulation of JA (A), OH-JA (B), JA-Ile (C), OH-JA-Ile (D), COOH-JA-Ile (E), ABA (F) and SA (G) was measured on LC-MS^3^. Statistically significant differences are indicated by asterisk (P < 0.05). Click here for file

Additional file 2: Table S1 Differentially regulated genes by NaMYC2 transcription factor in *N. attenuata.* Click here for file

Additional file 3: Table S2 Untargeted metabolomic analysis of herbivore induced metabolites of EV and MYC2. Click here for file

Additional file 4: Table S3 List of primers used for the experiments. Click here for file
