# Supplementary material for: NaMYC2 transcription factor regulates a subset of plant defense responses in Nicotiana attenuata
Source: BMC Plant Biol. 2013 May 1;13:73. doi: 10.1186/1471-2229-13-73 (PMC3655906; doi:10.1186/1471-2229-13-73)
Supplement: Additional file 1: Figure S1 — (A) Nucleotide sequences of NaMYC2 and NaMYC2-like (MYC2L) genes. (B) The relative accumulation (mean ± SE) of MYC2-like transcripts (MYC2L, n=5) was determined before (0 h) and after WOS treatment (1 h, 2 h) in EV (solid line) and MYC2-VIGS (dashed) N. attenuata plants by qRT-PCR. Figure S2. Alignment of nucleotide sequences of the N. attenuata MYC2 and MYC2-like TFs. Sequences were aligned by EMBOSS Stretcher program (http://www.ebi.ac.uk/Tools/psa/) and regions used for silencing of MYC2 and determination of transcript abundances were highlighted in color. Figure S3. (A) Control and WOS-treated leaves (n = 5) of EV and MYC2-VIGS plants were collected and used to analyze the accumulation (mean ± SE) of nicotine. (B) to (D): Leaves (n = 3) of EV and MYC2-VIGS plants attacked by neonates of M. sexta for 4 d were collected, extracted and analyzed for metabolites by HPLC-TOF-MS. The extracted ion chromatographs (EIC) for nicotine (B), anatabine (C) and cotinine (D) were overlaid to compare the regulation of alkaloid biosynthesis by MYC2. Figure S4. Secondary metabolite accumulations in EV and MYC2-VIGS plants before (0 h) or 24, 48 and 72 h after WOS treatment. Control and WOS-treated leaves (n = 3) of EV and MYC2-VIGS plants were collected and used to analyze the accumulation (mean ± SE) of caffeoylputrescine (A), dicaffeoylspermidine (B), chlorogenic acid (C), rutin (D) and total HGL-DTGs (E) on HPLC-PDA. Figure S5. Accumulation of phytohormones in EV and MYC2-VIGS plants. Fully elongated leaves of EV and MYC2-VIGS plants were treated with WOS and harvested after 1, 2, and 3 h, or collected without treatment. The accumulation of JA (A), OH-JA (B), JA-Ile (C), OH-JA-Ile (D), COOH-JA-Ile (E), ABA (F) and SA (G) was measured on LC-MS3. Statistically significant differences are indicated by asterisk (P < 0.05). [file 1471-2229-13-73-S1.pdf]

Supplemental Figures

NaMYC2 transcription factor regulates a subset of plant defense responses in *Nicotiana attenuata*

Woldemariam et al.

A

```
>MYC2
ATACACACACAAACACTTCGCTACCCAAACAAACTCTCTCCATTTTCACTC
ACTCCTTATCACCAAACAATTCTTGGGTTTTTGAATATATACCCTAAAAAGTTCCTCTC
CATTTTCTCTCTCTGTATCAAGAATCAAACAGATCTGAATTGATTTATCTGTTTTTTTC
TTGTTTTTTGTTATATGGAATGACGGACTATAGAATACCAACGATGAATAATATATGGAG
CAATACTACATCAGACGATAACATGATGGAAGCTTTTTTATCTTCTGATCCGTCGTCGTT
TTGGCCCGGAACAACTACTACACCAACTCCCCGGACTTCAGTTTCTCCGGCGCCGCCGCC
GGTGACGAGTATTGCCGGAGACCCATTAAAGTCTATGCCATATTTTAACCAAGAGTCGCT
GCAACAGCGACTTCAGACTTTAATCGACGGGGCTCGTGAAGCGTGGACGTATGCCATATT
TTGGCAATCGTCTGTTGTGGATTTCGCGAGCCCCTCTGTGTTGGGGTGGGGAGATGGGTA
TTATAAAGGTGAAGAAGATAAAAATAAGCGTAAAACGGCGTCGTTTTCGCCTGATTTTAT
CACGGAGCAAGCACACCGGAAAAAGGTTCTCCGGGAGCTGAATTCTTTAATTTCCGGCAC
ACAAACTGGTGGTGAAAATGATGCTGTCGATGAAGAAGTAACGGATACTGAATGGTTTTT
TCTGATTTCAATGACTCAATCGTTTGTTAACGGAAGCGGGCTTCCGGGCCTGGCGATGTA
CAGCTCAAGCCCGATTTGGGTTACTGGAGCAGAGAGATTAGCTGCTTCGCACTGCGAACG
GGCCCGACAAGCCCAAGGATTCGGGCTTCAGACTATTGTTTGTATTCCTTCAGCTAATGG
TGTTGTTGAGCTCGGGTCAACTGAGTTGATATTCCAGACTGCTGATTTAATGAACAAGGT
TAAAGTTTTGTTTAATTTTAATATTGATATGGGTGCGACTACGGGCTCAGGATCGGGCTC
ATGTGCTATTCACGCTGAGCCCGATCCTTCGGCCCTTTGGCTGACGGATCCGGCTTCCTC
AGCTGTGGAAGTCAAGGATTCGAATACAGTTCCTTCAAGTAATAGTAGTAAGCAACTTGT
GTTTGGAAATGAGAATTCCGAAAATGGTAATCAAAATTCTCAGCAAACACAAGGATTTTT
CACTAGGGAGTTGAATTTTTCCGAATATGGATTTGATGGAAGTAATACTCGGAATGGGAA
TGCAAATTCTTCGCGTTCTTGCAAGCCCGAGTCTGGTGAAATCTTGAATTTTGGTGATAG
TACTAAGAGAAGTGCTTCAAGTGCAAATGGGAGCTTGTTTTCGGGCCAATCACAGTTCGG
GCCCGGGTCTGCGGAGGAGAACAAGAACAAGAACAAGAAAAGGTCACCTGCATCAAGAGG
TAGCAACGATGAAGGGATGCTTTCATTTGTTTCGGGTGTGATCTTGCCAAGTTCAAACAC
GGGGAAGTCCGGTGGAGGTGGCGATTCGGATCAATCAGATCTCGAGGCTTCGGTGGTGAA
GGAGGCGGATAGTAGTAGAGTTGTAGACCCTGAGAAGAAGCCGAGGAAACGAGGGAGGAA
ACCCGCTAACGGGAGAGAGGAGCCGTTGAATCATGTGGAGGCAGAGAGACAAAGGAGGGA
GAAATTAAATCAAAGATTCTATGCACTTAGAGCTGTTGTACCAAATGTGTCAAAAATGGA
TAAAGCATCACTTCTTGGTGATGCAATTGCATTTATCAATGAGTTGAAATCAAAGGTTCA
GAATTCTGACTCAGATAAAGAAGAGTTGAGGAACCAAATTGAATCTTTAAGGAAAGAATT
AGCCAACAAGGGATCAAACTATACCGGTCCTCCACCGTCAAATCAAGATCTCAAAATTCT
AGATATGGATATCGACGTTAAGGTGATCGGATGGGATGCTATGATTCGTATACAATCTAA
TAAAAAGAACCATCCAGCCGCGAGGTTAATGGCCGCTCTCATGGAATTGGACTTAGATGT
GCACCATGCTAGTGTTTCAGTGGTCAACGAGTTGATGATCCAACAAGCAACTGTGAAAAT
GGGGAGTCGGCTTTACACGCAAGAACAACTCCGGATATCATTGACATCCAGAATTGCTGA
ATCGCGATGAAGAGAAATACAGTAAATGGAAATTATCATAGTGAGCTCTGAATAATGTTA
TCTTTCATTGAGCTATTTTAAGAGAATTTCTCCTATAAGTTAGATCTTGAGTTTAAGGCT
ACTTAAAGTGGAAAGCTGATTGAGCTTTCCTCTTAGTTTTTGGGTATTTTTCAACTTTTA
TATCTAGTTTGTTTTCCACATTTTCTGTACATAAATGTGAAACCAATACTAGATCTCAAG
TTATGCTTTTTAGTTCATGTAATTAGAAATAAATATGCAGTTTCATCTTTTTCTGTTAAG
GTCTGCTCCATCTATTTGTATTCTTTATCCAGAATA
```

```
>MYC2L
ATCTTTCTACCTTCCGCTACCTTCTCTCTCCG
CCGCACACCCTCTTTTCACCCATTATTCTCTCTCTCTATATATATATCTATATATCTTTC
ACGCCACCACTTCCAACTGTTTGTGCTGGGTTTATGGACTGACTGATTACAGCTTACCCA
CCATGAATTTGTGGAATACTAGTGGTACTACCGATGACAACGTTTCTATGATGGAAGCTT
TTATGTCTTCTGATCTCACTTCATTTTGGGCTACTTCTAATTCTACTGCTGCTGCTGTTA
CCTCTAATTCTGATCATATTCCAGTTAATACCCCAACGGTTCTTCTTCCGTCTTCTTGTG
CTTCTACTGTCACAGCTGTGGCTGTCGATGCTTCAAAATCCATGCCTTTTTTCAACCAAG
AAACCCTTCAACAGCGTCTTCAAACCCTCATTGATGGTGCTCGCGAGACATGGACCTATG
CCATCTTTTGGCAGTCATCCGTCGTTGATTTAACGAGTCCATTTGTGTTGGGCTGGGGAG
ATGGTTACTACAAAGGTGAAGAAGATAAAGCCGGTCGGAAATTAGCTGTTTCTTCTCCTG
CTTATATAGCTGAGCAAGAACACCGGAAAAAGGTTCTCCGGGAGCTGAATTCGTTGATCT
CCTGCACGCAAACGGGCACTGATGATGCCGTCGATGAAGAAGTTACCGACACTGAATGGT
TCTTCCTTATTTCCATGACCCAATCGTTTGTTAACGGAAGTGGGCTTCCGGGTCAGGCCT
TATACAATTCCAGCCCCATTTGGGTAGCCGGAGCAGAGAAATTGGCAGCTTCCCACTGCG
AACGGGCTCGGCAGGCCCAGGGATTCGGGCTTCAGACGATGGTTTGTATTCCTTCAGCAA
ACGGCGTGGTTGAATTGGGCTCCACGGAGTTGATTATTCAGAGTTCTGATCTCATGAACA
AGGTTAGAGTATTGTTTAACTTCAATAATGATTTGGGCTCTGGTTCGTGGGCTGTGCAGC
CCGAGAGCGACCCGTCCGCTCTTTGGCTCACTGATCCATCGCCTGCAGCTGTACAAGTCA
AAGATTTAAATACAGTTCCATCAAGTAATAGTAGTAAGCAAGTTGTGTTTGATAATGAGA
ATAATGGTCACATTTGTGATAATCAGCAACAGCACCATTCTCAGCAACAAACACAAGGAT
TTTTCACAAGGGAGTTGAACTTTTCAGAATTCGGGTTTGATGGAAGTAGTAATAATAGGA
ATGGGAATTCATCAGTTTCTTGCAAGCCAGAGTCGGGGGAAATCTTGAATTTTGGTGATA
GCACTAAGAAAAGTGCAAATGGGAACTTATTTTCGGGTCAGTCCCATTTTGGGGCAGGGG
AGGAGAATAAGAACAAGAAAAGGTCACCTGCTTCCAGAGGAAGCAATGAAGAAGGAATGC
TTTCATTTGTTTCAGGTACAATCTTGCCTGCAGCTTCTGGTGCGATGAAGTCAAGTGGAT
GTGTCGGTGAAGACTCCTCTGATCATTCGGATCTTGAGGCCTCAGTGGTGAAAGAAGCTG
AAAGTAGTAGAGTTGTAGAACCCGAAAAGAGGCCAAAGAAGCGAGGAAGGAAGCCAGCAA
ATGGACGTGAGGAACCTTTGAATCACGTCGAAGCAGAGAGGCAAAGGAGAGAGAAATTAA
ACCAAAGGTTCTACGCTTTAAGAGCTGTTGTTCCGAATGTGTCCAAGATGGACAAGGCAT
CACTGCTTGGAGATGCAATTTCATATATTAATGAGCTGAAGTTGAAGCTTCAAAATACAG
AAACAGATAGAGAAGACTTGAAGAGCCAAATAGAAGATTTGAAGAAAGAATTAGCTAGTG
AAGACTCATGGCGCCCTGGTCCTCCACCAAATCAAGATCACAAGATGTCTAGCCATACTG
GAAGCAAGATTGTAGATGTGGATATAGATGTTAAGATAATTGGATGGGATGCGATGGTTC
GTATACAATGTAATAAAAAGAACCATCCAGCTGCAAGGTTAATGGTAGCCCTCAAGGAGT
TAGATCTAGAAGTGCACCATGCCAGTGTTTCAGTGGTGAACGATTTGATGATCCAACAAG
CCACAGTGAAAATGGGTAGCAGACTTTACACGGAAGAGCAACTTAGGATAGCATTGACAT
CCAGAGTTGCTGAAACACGCTAAAAACACTTGAGATCTCAATTTGTAGGCTTTGAGTTAG
CCTTGTAAATTGTGTTCCAGACTATGCTAAATTTAAGAGCTCTGTCTAATGTTTTTGTCA
TCAAGTTAGAGATTAAGATCAAGGCTCTTTTTGTGTTATTTTTCCACTTGTACCTAGGAA
TATTCGTGTACATGATTGTTAAAAAAAAAGTCGGGTGGTAGTTCTAGTTCATGTAAAAAT
TTGCAGCTCCATCCTCTCCTTCAGTATTCTTTGTTCAGGATATATATATGAAGAAGAAAT
TGATGGTTGGT
```

B

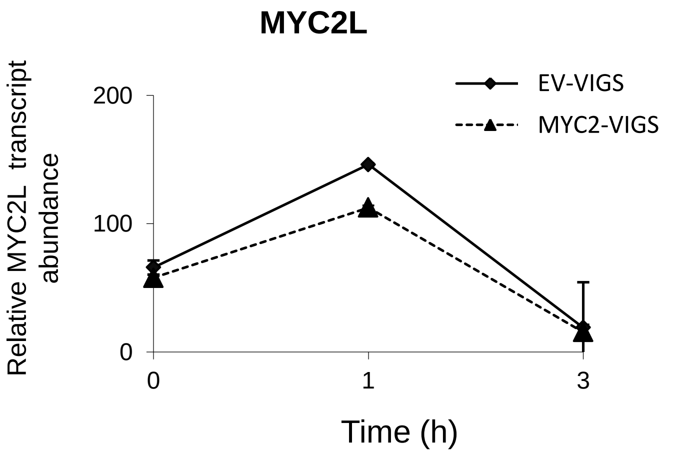

Figure S1. Nucleotide sequences and transcript abundances of MYC2-like transcription factor in MYC2-VIGS silenced *N. attenuata* plants. (A) Nucleotide sequences of *MYC2* and *MYC2-like* (*MYC2L*) genes in *N. attenuata*. (B) Transcript accumulation of NaMYC2 transcription factors was suppressed by Virus Induced Gene Silencing (VIGS; Fig. 2C) and the relative mean ± SE accumulation of *MYC2-like* transcripts (*MYC2L*, n=5) were determined before (0 h) and after WOS treatment (1 h, 2 h) in EV (solid line) and MYC2-VIGS (dashed) plants by qRT-PCR. GenBank Accession numbers: NaMYC2 (KC832837); NaMYC2-like (KC906192).

```
MYC2               1 ATACACACACAAACACTTCGCTACCCAAACAAACTCTCTCCATTTTCACT     50
                     || |...|.   ||..|.|||||||..      ||||||||.... |||.
MYC2L              1 AT-CTTTCT---ACCTTCCGCTACCTT------CTCTCTCCGCCG-CACA     39

MYC2              51 CACTCCTTATCACCAAACAATTCTTGGGTTTTTGAATATATACCCTAAAA    100
                     |.||| ||.||||    |.|||.||...|.|.|..|||||||
MYC2L             40 CCCTC-TTTTCAC----CCATTATTCTCTCTCTATATATA--------     76

MYC2             101 AAGTTCCTCTCCATTTTCTCTCTCTGTATCAAGAATCAAACAGATCTGAA    150
                                   |.|||.|.|.|.|.|||.|   |.|.||..||. ||
MYC2L             77 --------------TATCTATATATCTTTCACG---CCACCACTTCC-AA    108

MYC2             151 TTGATTTATCTGTTTTTTTTCTTGTTTTTTGTTATATGGAATGACGGACT    200
                     .||           |||.|.||.|.||       ||||||.||||.||.|
MYC2L            109 CTG-----------TTTGTGCTGGGTT-------TATGGACTGACTGATT    140

MYC2             201 ATAGAATACCAACGATGAATAATATATGGAGCAATACT---ACATCAGAC    247
                     |.||..||||.||.||||||   .|.||||..|.||.|   ||..|.||.
MYC2L            141 ACAGCTTACCCACCATGAAT---TTGTGGAATACTAGTGGTACTACCGAT    187

MYC2             248 GATAAC------ATGATGGAAGCTTTTTTATCTTCTGATCCGTCGTCGTT    291
                     ||.|||      |||||||||||||||.|.||||||||||...|.||.||
MYC2L            188 GACAACGTTTCTATGATGGAAGCTTTTATGTCTTCTGATCTCACTTCATT    237

MYC2             292 TTGGCCCGGAACAACTACTACACCAACTCCCCGGACTTCAGTTTCT----    337
                     ||||.|.....|.|.|.||||..|..||.|....||.||...||||
MYC2L            238 TTGGGCTACTTCTAATTCTACTGCTGCTGCTGTTACCTCTAATTCTGATC    287

MYC2             338 -----CCGG------CGCCGCCG---------CCG---------------    352
                          ||.|      |.||..||         |||
MYC2L            288 ATATTCCAGTTAATACCCCAACGGTTCTTCTTCCGTCTTCTTGTGCTTCT    337

MYC2             353 ---GTGACGAGTATTGCCGGAGACCCATTAAAGTCTATGCCATATTTTAA    399
                        ||.||...|.|.||.|..||..|.|.|||.||.|||||.|.|||.||
MYC2L            338 ACTGTCACAGCTGTGGCTGTCGATGCTTCAAAATCCATGCCTTTTTTCAA    387

MYC2             400 CCAAGAGTCGCTGCAACAGCGACTTCAGACTTTAATCGACGGGGCTCGTG    449
                     ||||||..|.||.||||||||.|||||.||..|.||.||.||.|||||.|
MYC2L            388 CCAAGAAACCCTTCAACAGCGTCTTCAAACCCTCATTGATGGTGCTCGCG    437

MYC2             450 AAGCGTGGACGTATGCCATATTTTGGCAATCGTCTGTTGTGGATTTCGCG    499
                     |..|.|||||.||||||||.||||||||.||.||.||.||.|||||..||
MYC2L            438 AGACATGGACCTATGCCATCTTTTGGCAGTCATCCGTCGTTGATTTAACG    487

MYC2             500 AGCCCCTCTGTGTTGGGGTGGGGAGATGGGTATTATAAAGGTGAAGAAGA    549
                     ||.||.|.|||||||||.|||||||||||.||.||.||||||||||||||
MYC2L            488 AGTCCATTTGTGTTGGGCTGGGGAGATGGTTACTACAAAGGTGAAGAAGA    537

MYC2             550 TAAAAATAAGCGTAAAACGGCGTCGTTTTCGCCTGATTTTATCACGGAGC    599
                     ||||......||.|||...||....|.|||.||||.||.|||..|.||||
MYC2L            538 TAAAGCCGGTCGGAAATTAGCTGTTTCTTCTCCTGCTTATATAGCTGAGC    587

MYC2             600 AAGCACACCGGAAAAAGGTTCTCCGGGAGCTGAATTCTTTAATTTCCGGC    649
                     |||.|||||||||||||||||||||||||||||||||.||.||.|||.||
MYC2L            588 AAGAACACCGGAAAAAGGTTCTCCGGGAGCTGAATTCGTTGATCTCCTGC    637

MYC2             650 ACACAAACTGGTGGTGAAAATGATGCTGTCGATGAAGAAGTAACGGATAC    699
                     ||.|||||.||...||   |||||||.||||||||||||||.||.||.||
MYC2L            638 ACGCAAACGGGCACTG---ATGATGCCGTCGATGAAGAAGTTACCGACAC    684
```

```
MYC2             700 TGAATGGTTTTTTCTGATTTCAATGACTCAATCGTTTGTTAACGGAAGCG  749
                     |||||||||.||.||.|||||.|||||.||||||||||||||||||||.|
MYC2L            685 TGAATGGTTCTTCCTTATTTCCATGACCCAATCGTTTGTTAACGGAAGTG  734

MYC2             750 GGCTTCCGGGCCTGGCGATGTACAGCTCAAGCCCGATTTGGGTTACTGGA  799
                     ||||||||||.|.|||..|.||||..||.|||||.||||||||..|.|||
MYC2L            735 GGCTTCCGGGTCAGGCCTTATACAATTCCAGCCCCATTTGGGTAGCCGGA  784

MYC2             800 GCAGAGAGATTAGCTGCTTCGCACTGCGAACGGGCCCGACAAGCCCAAGG  849
                     |||||||.|||.||.|||||.||||||||||||||.||.||.|||||.||
MYC2L            785 GCAGAGAAATTGGCAGCTTCCCACTGCGAACGGGCTCGGCAGGCCCAGGG  834

MYC2             850 ATTCGGGCTTCAGACTATTGTTTGTATTCCTTCAGCTAATGGTGTTGTTG  899
                     |||||||||||||||.||.|||||||||||||||||.||.||.||.||||
MYC2L            835 ATTCGGGCTTCAGACGATGGTTTGTATTCCTTCAGCAAACGGCGTGGTTG  884

MYC2             900 AGCTCGGGTCAACTGAGTTGATATTCCAGACTGCTGATTTAATGAACAAG  949
                     |..|.||.||.||.||||||||..|.||||.|.|||||.|.|||||||||
MYC2L            885 AATTGGGCTCCACGGAGTTGATTATTCAGAGTTCTGATCTCATGAACAAG  934

MYC2             950 GTTAAAGTTTTGTTTAATTTTAATATTGATATGGGTGCGACTACGGGCTC  999
                     ||||.|||.||||||||.||.||||.||||.||||            |||
MYC2L            935 GTTAGAGTATTGTTTAACTTCAATAATGATTTGGG------------CTC  972

MYC2            1000 AGGATCGGGCTCATGTGCTATTCACGCTGAGCCCGATCCTTCGGCCCTTT 1049
                     .||.|||      ||.|||.|.||..|.|||..|||.||.||.||.||||
MYC2L            973 TGGTTCG------TGGGCTGTGCAGCCCGAGAGCGACCCGTCCGCTCTTT 1016

MYC2            1050 GGCTGACGGATCCGGCTTCCTCAGCTGTGGAAGTCAAGGATTCGAATACA 1099
                     ||||.||.|||||..|..|..|||||||..|||||||.||||..||||||
MYC2L           1017 GGCTCACTGATCCATCGCCTGCAGCTGTACAAGTCAAAGATTTAAATACA 1066

MYC2            1100 GTTCCTTCAAGTAATAGTAGTAAGCAACTTGTGTTTGGAAATGAGAATTC 1149
                     |||||.|||||||||||||||||||||.||||||||..||||||||||..
MYC2L           1067 GTTCCATCAAGTAATAGTAGTAAGCAAGTTGTGTTTGATAATGAGAATAA 1116

MYC2            1150 CGAAAA------TGGTAATCA--AA-------ATTCTCAGCAA---ACAC 1181
                     .|...|      ||.||||||  ||       |||||||||||   ||||
MYC2L           1117 TGGTCACATTTGTGATAATCAGCAACAGCACCATTCTCAGCAACAAACAC 1166

MYC2            1182 AAGGATTTTTCACTAGGGAGTTGAATTTTTCCGAATATGGATTTGATGGA 1231
                     |||||||||||||.|||||||||||.|||||.||||..||.|||||||||
MYC2L           1167 AAGGATTTTTCACAAGGGAGTTGAACTTTTCAGAATTCGGGTTTGATGGA 1216

MYC2            1232 AGTAATACTCGGAATGGGAATGCAAATTCTTCGCGTTCTTGCAAGCCCGA 1281
                     ||||.||.|   |||.||||||..|||||.||...|||||||||||||.||
MYC2L           1217 AGTAGTAAT---AATAGGAATGGGAATTCATCAGTTTCTTGCAAGCCAGA 1263

MYC2            1282 GTCTGGTGAAATCTTGAATTTTGGTGATAGTACTAAGAGAAGTGCTTCAA 1331
                     |||.||.|||||||||||||||||||||||.|||||||.         ||
MYC2L           1264 GTCGGGGGAAATCTTGAATTTTGGTGATAGCACTAAGAA---------AA 1304

MYC2            1332 GTGCAAATGGGAGCTTGTTTTCGGGCCAATCACAGTTCGGGCCCGGGTCT 1381
                     ||||||||||||.|||.||||||||.||.||.||.||.|||.|.|||
MYC2L           1305 GTGCAAATGGGAACTTATTTTCGGGTCAGTCCCATTTTGGGGCAGGG--- 1351

MYC2            1382 GCGGAGGAGAACAAGAACAAGAACAAGAAAAGGTCACCTGCATCAAGAGG 1431
                        ||||||||.||||||||      |||||||||||||||.||.|||||
MYC2L           1352 ---GAGGAGAATAAGAACAA------GAAAAGGTCACCTGCTTCCAGAGG 1392
```

```
MYC2        1432 TAGCAACGATGAAGGGATGCTTTCATTTGTTTCGGGTGTGATCTTGCC--   1479
                 .|||||.||.|||||.||||||||||||||||.|||...||||||||
MYC2L       1393 AAGCAATGAAGAAGGAATGCTTTCATTTGTTTCAGGTACAATCTTGCCTG   1442

MYC2        1480 -AAGTTCAAACACGGGGAAGTCCGGTGGAGGTGGCGAT---------TCG   1519
                  |..|||.....||..||||||..|||||.|||.||.|         ||.
MYC2L       1443 CAGCTTCTGGTGCGATGAAGTCAAGTGGATGTGTCGGTGAAGACTCCTCT   1492

MYC2        1520 GATCAATCAGATCTCGAGGCTTCGGTGGTGAAGGAGGCGGATAGTAGTAG   1569
                 |||||.||.|||||.|||||.||.||||||||.||.||.||.||||||||
MYC2L       1493 GATCATTCGGATCTTGAGGCCTCAGTGGTGAAAGAAGCTGAAAGTAGTAG   1542

MYC2        1570 AGTTGTAGACCCTGAGAAGAAGCCGAGGAAACGAGGGAGGAAACCCGCTA   1619
                 |||||||||.||.||.||||.|||.|.|||.|||||.|||||.||.||.|
MYC2L       1543 AGTTGTAGAACCCGAAAAGAGGCCAAAGAAGCGAGGAAGGAAGCCAGCAA   1592

MYC2        1620 ACGGGAGAGAGGAGCCGTTGAATCATGTGGAGGCAGAGAGACAAAGGAGG   1669
                 |.||..|.|||||.||.||||||||.||.||.||||||||.||||||||.
MYC2L       1593 ATGGACGTGAGGAACCTTTGAATCACGTCGAAGCAGAGAGGCAAAGGAGA   1642

MYC2        1670 GAGAAATTAAATCAAAGATTCTATGCACTTAGAGCTGTTGTACCAAATGT   1719
                 |||||||||||.|||||.|||||.||..|.|||||||||||.||.|||||
MYC2L       1643 GAGAAATTAAACCAAAGGTTCTACGCTTTAAGAGCTGTTGTTCCGAATGT   1692

MYC2        1720 GTCAAAAATGGATAAAGCATCACTTCTTGGTGATGCAATTGCATTTATCA   1769
                 |||.||.|||||.||.||||||||.|||||.|||||||||.|||.|||.|
MYC2L       1693 GTCCAAGATGGACAAGGCATCACTGCTTGGAGATGCAATTTCATATATTA   1742

MYC2        1770 ATGAGTTGAAATCAAAGGTTCAGAATTCTGACTCAGATAAAGAAGAGTTG   1819
                 |||||.||||.|..|||.||||.|||.|.||..||||||.||||||.|||
MYC2L       1743 ATGAGCTGAAGTTGAAGCTTCAAAATACAGAAACAGATAGAGAAGACTTG   1792

MYC2        1820 AGGAACCAAATTGAATCTTTAAGGAAAGAATTAGCCAACAAGGGATCAAA   1869
                 |.||.||||||.|||..|||.|.||||||||||||.|...|.|..|||..
MYC2L       1793 AAGAGCCAAATAGAAGATTTGAAGAAAGAATTAGCTAGTGAAGACTCATG   1842

MYC2        1870 CTATACCGGTCCTCCACCG--TCAA-ATCA--AGATCTC-----------   1903
                 .....|.|||||||||||.  |||| ||||  ||||.||
MYC2L       1843 GCGCCCTGGTCCTCCACCAAATCAAGATCACAAGATGTCTAGCCATACTG   1892

MYC2        1904 -----AAAATTCTAGATATGGATATCGACGTTAAGGTGATCGGATGGGAT   1948
                      ||.|||.|||||.|||||||.||.||||||.|.||.|||||||||
MYC2L       1893 GAAGCAAGATTGTAGATGTGGATATAGATGTTAAGATAATTGGATGGGAT   1942

MYC2        1949 GCTATGATTCGTATACAATCTAATAAAAAGAACCATCCAGCCGCGAGGTT   1998
                 ||.|||.||||||||||||.||||||||||||||||||||.||.|||||
MYC2L       1943 GCGATGGTTCGTATACAATGTAATAAAAAGAACCATCCAGCTGCAAGGTT   1992

MYC2        1999 AATGGCCGCTCTCATGGAATTGGACTTAGATGTGCACCATGCTAGTGTTT   2048
                 |||||..||.||||.|||.||.||..||||.|||||||||||.|||||||
MYC2L       1993 AATGGTAGCCCTCAAGGAGTTAGATCTAGAAGTGCACCATGCCAGTGTTT   2042

MYC2        2049 CAGTGGTCAACGAGTTGATGATCCAACAAGCAACTGTGAAAATGGGGAGT   2098
                 |||||||.|||||.|||||||||||||||||.||.||||||||||||.||.
MYC2L       2043 CAGTGGTGAACGATTTGATGATCCAACAAGCCACAGTGAAAATGGGTAGC   2092

MYC2        2099 CGGCTTTACACGCAAGAACAACTCCGGATATCATTGACATCCAGAATTGC   2148
                 .|.|||||||||.||||.|||||..|||||.||||||||||||||.||||
MYC2L       2093 AGACTTTACACGGAAGAGCAACTTAGGATAGCATTGACATCCAGAGTTGC   2142
```

```
MYC2            2149 TGAATCGCGATGAAGAGAAAT-ACAG-TAAATGGAAATTATCATAGTGAG   2196
                     ||||.|.||.|.||.|.|..| |.|. |.|||....|...|...|||.||
MYC2L           2143 TGAAACACGCTAAAAACACTTGAGATCTCAATTTGTAGGCTTTGAGTTAG   2192

MYC2            2197 CTCTGAATAATGTTATCTTTCATTGAGCTAT-TTTAAGAGAATTTCTCCT   2245
                     |..||.|.|.|||..||... |.|..||||. ||||||||. |.|.||..
MYC2L           2193 CCTTGTAAATTGTGTTCCAG-ACTATGCTAAATTTAAGAGC-TCTGTCTA   2240

MYC2            2246 ATAAGTTAGATCTTGAGTTTAAGGCTACTTAAAGTGGAAAGCTGATTGAG   2295
                     ||      |.|.|||...|.|||     |||.||...||    |||..||
MYC2L           2241 AT------GTTTTTGTCATCAAG-----TTAGAGATTAA----GATCAAG   2275

MYC2            2296 CTTTCCTCTTAGTTTTTGGGTATTTTTCAACTTTTATATCTAGTTTGTTT   2345
                     .    |||||   |||..|.||||||||.||||.||  .||||..
MYC2L           2276 G----CTCTT---TTTGTGTTATTTTTCCACTTGTA--CCTAGGA-----   2311

MYC2            2346 TCCACATTTTC-TGTACATAAATGTGAAACCAATACTAGATCTCAAGTTA   2394
                          ||.||| |||||||.|.|||.|||..||.|.|.|......|||
MYC2L           2312 -----ATATTCGTGTACATGATTGTTAAAAAAAAAGTCGGGTGGTAGT--   2354

MYC2            2395 TGCTTTTTAGTTCATGTAATTAGAAATAAATATGCAGTTTCATCTTTTTC   2444
                          |.||||||||||||        ||||.|||||.|.||||.|.|.|
MYC2L           2355 -----TCTAGTTCATGTAA--------AAATTTGCAGCTCCATCCTCTCC   2391

MYC2            2445 TGT--TAAGGTCTGCTCC----ATCTATTTGTATTCTTTATCCAGAATA-   2487
                     |..  ||...|.||.||.    ||.|||.||.|......||..|...|.
MYC2L           2392 TTCAGTATTCTTTGTTCAGGATATATATATGAAGAAGAAATTGATGGTTG   2441

MYC2            2488 --    2487

MYC2L           2442 GT    2443
```

Red highlighted sequence was used for MYC2 silencing by VIGS

MYC2 primer pair is **underlined in bold blue**

MYC2L primer pair is **underlined in bold green**

Figure S2. Nucleotide alignment of MYC2 (KC832837) and MYC2-Like (MYC2L; KC906192) transcription factors from *N. attenuata* plants. The nucleotide sequences were aligned by EMBOSS Stretcher program (http://www.ebi.ac.uk/Tools/psa/). Sequence regions used for silencing of *MYC2* gene in MYC2-VIGS plants and qRT-PCR determination of transcript abundances were highlighted in color.]

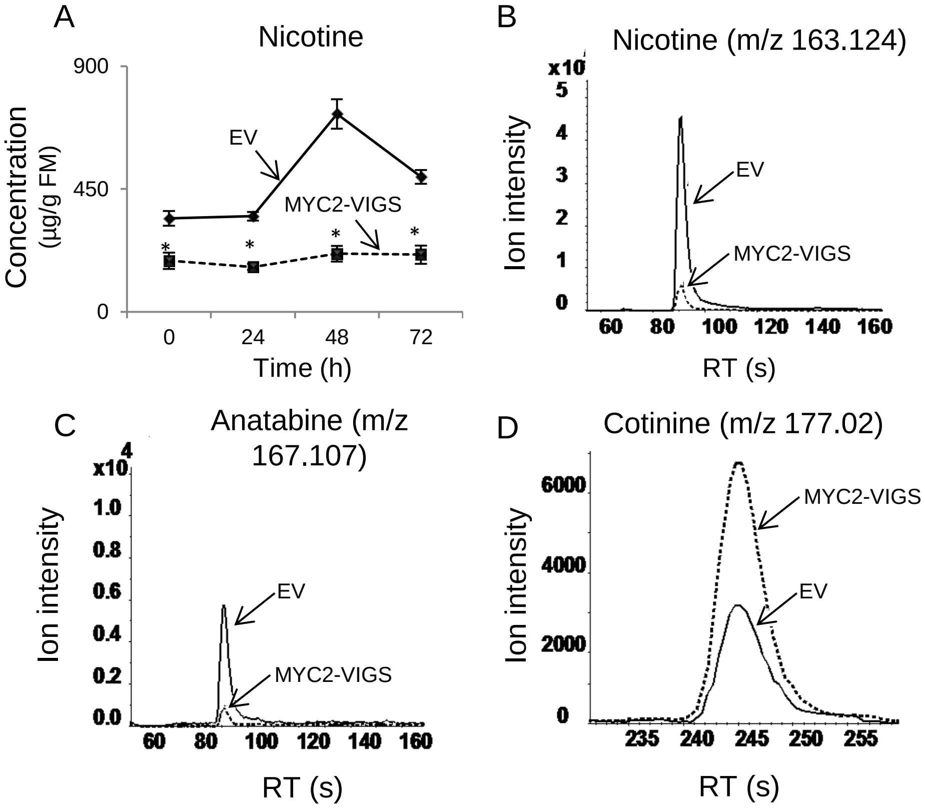

Figure S3. **Targeted and untargeted analysis of nicotine and related alkaloids in EV and MYC2-VIGS plants.** (A) Control and WOS-treated leaves (n = 5) of EV and MYC2-VIGS plants were collected and used to analyze the accumulation (mean ± SE) of nicotine. Statistically significant differences are indicated by asterisk. (B) to (D). Leaves (n = 3) of EV and MYC2-VIGS plants that were attacked by neonates of *M. sexta* for 4 d were collected to extract metabolites which were subsequently analyzed by HPLC-TOF-MS. The extracted ion chromatographs (EIC) for nicotine (B), anatabine (C) and cotinine (D) were overlaid to compare the regulation of alkaloid biosynthesis by MYC2.

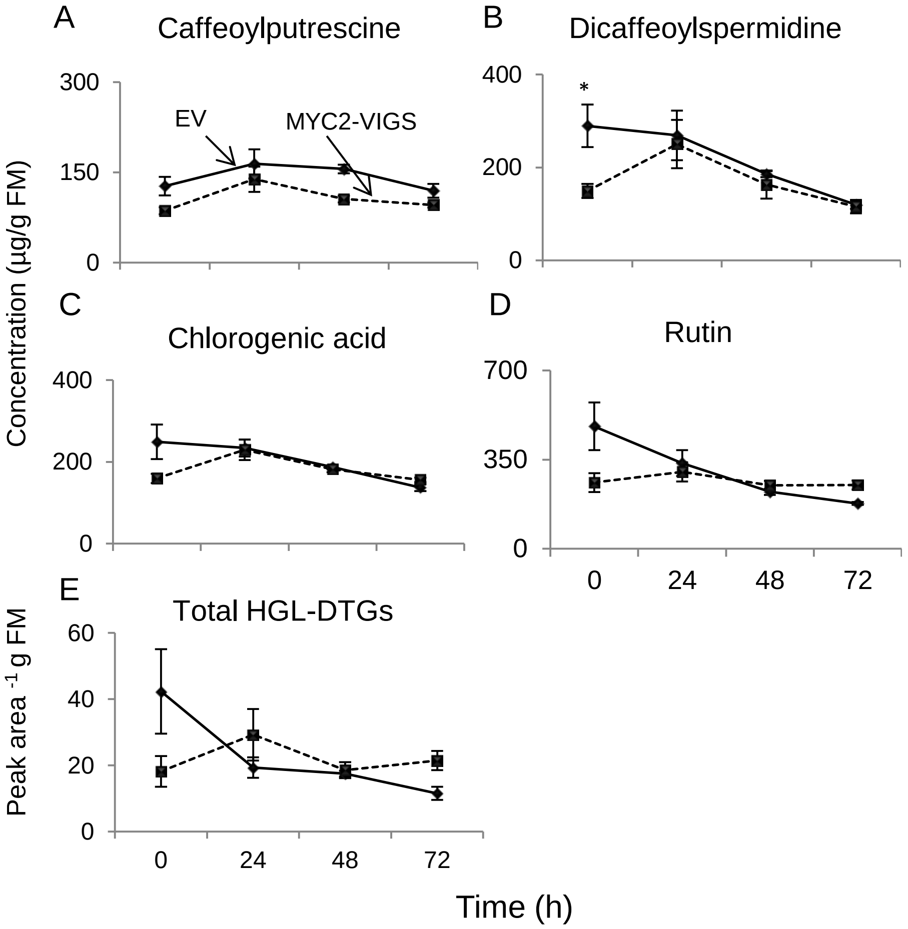

Figure S4. **Secondary metabolites accumulation in EV and MYC2-VIGS plants before (0 h) or 24 , 48 and 72 h after WOS treatment**. Control and WOS-treated leaves (*n* = 3) of EV and MYC2-VIGS plants were collected and used to analyze the accumulation (mean ± SE) of caffeoylputrescine (A), dicaffeoylspermidine (B), chlorogenic acid (C), rutin (D) and total HGL-DTGs (E) on HPLC-PDA.

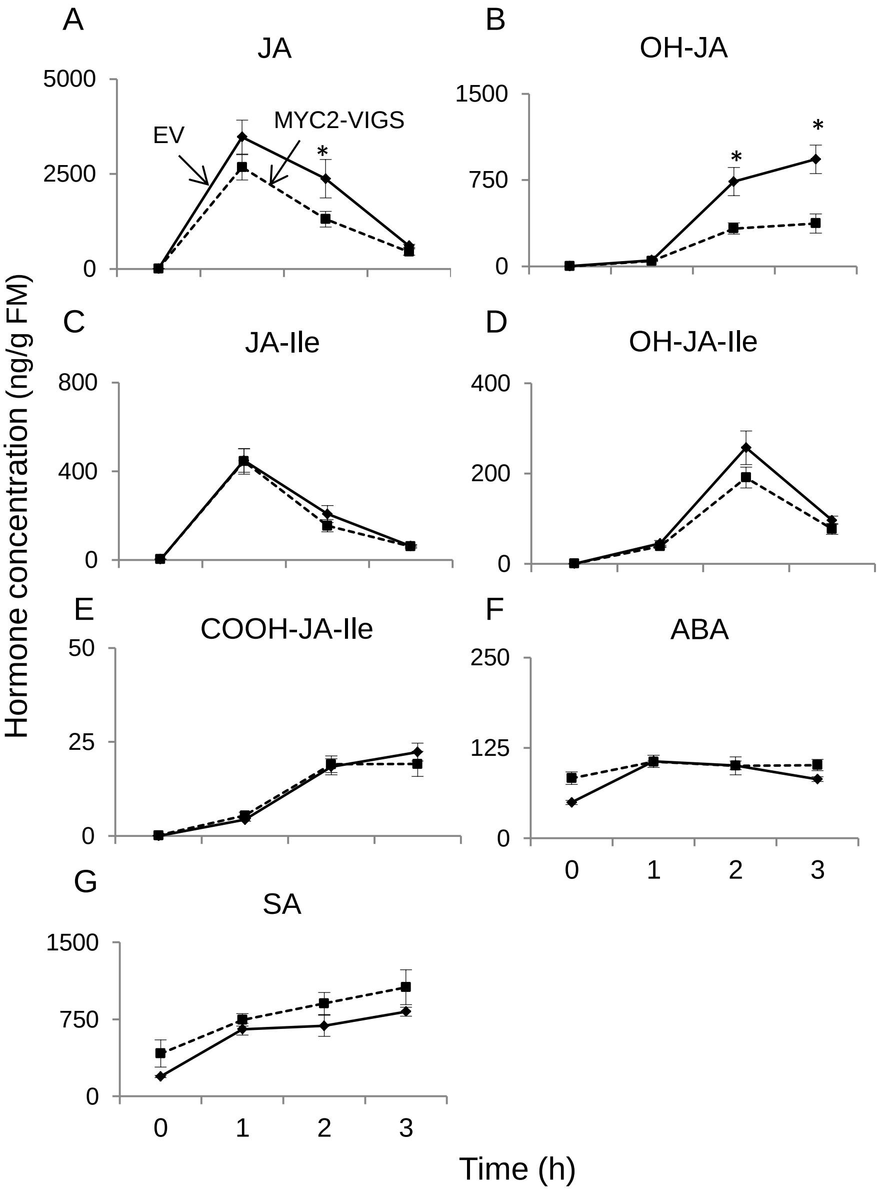

Figure S5. **Accumulation of phytohormones in untreated and WOS-treated EV and MYC2-VIGS plants**. Fully elongated leaves of EV and MYC2-VIGS plants were treated with WOS after 1, 2 , and 3 h or collected without treatment and the accumulation of JA (A), OH-JA (B), JA-Ile (C), OH-JA-Ile (D), COOH-JA-Ile (E), ABA (F) and SA (G) was measured on LC-MS[3]. Differences that are statistically significant are indicated by asterisk ($P < 0.05$).
