# Supplementary material for: NaMYC2 transcription factor regulates a subset of plant defense responses in Nicotiana attenuata
Source: BMC Plant Biol. 2013 May 1;13:73. doi: 10.1186/1471-2229-13-73 (PMC3655906; doi:10.1186/1471-2229-13-73)
Supplement: Additional file 2: Table S1 — Differentially regulated genes by NaMYC2 transcription factor in N. attenuata. [file 1471-2229-13-73-S2.docx]

**Table S1. Differentially regulated genes by NaMYC2 transcription factor in *N. attenuata.***

a. List of down-regulated genes in MYC2-VIGS plants

| **Probe ID** | **Fold change** | **BIN CODE** | **TAIR No** | **Name** | **Description** |
| --- | --- | --- | --- | --- | --- |
| Na_454_35716 | 2.31 | 10.6 | at5g62150 | cell wall.degradation | peptidoglycan-binding LysM domain-containing protein |
| Na_454_11932 | 3.60 | 13.1.1.1.1 | at5g17330 | amino acid metabolism | GAD; calmodulin binding / glutamate decarboxylase |
| Na_454_38855 | 4.52 | 13.1.4.4.1 | at1g74040 | amino acid metabolism | IMS1, MAML-3, IPMS2 \| IMS1 (2-ISOPROPYLMALATE SYNTHASE 1) |
| Na_454_02419 | 3.82 | 13.2.3.1.1 | at3g16150 | amino acid metabolism.degradation | L-asparaginase |
| Na_454_34394 | 3.75 | 13.2.3.1.1 | at3g16150 | amino acid metabolism.degradation | L-asparaginase, putative / L-asparagine amidohydrolase |
| Na_454_33674 | 4.58 | 13.2.4.4 | at3g45300 | amino acid metabolism.degradation | IVD (ISOVALERYL-COA-DEHYDROGENASE |
| Na_454_16864 | 2.69 | 16.1.5 | at2g24210 | secondary metabolism.isoprenoids.terpenoids | TPS10 (terpene synthase 10 ; (E-beta-ocimene synthase/ myrcene synthase) |
| Na_454_41485 | 9.09 | 16.1.5 | at3g25810 | secondary metabolism.isoprenoids.terpenoids | myrcene/ocimene synthase, putative |
| Na_454_26468 | 2.34 | 20.1.3.1 | at4g02600 | stress.biotic.signalling.MLO-like | ATMLO1 \| MLO1; calmodulin binding |
| Na_454_11776 | 4.18 | 20.1.7 | at1g69550 | stress.biotic.PR-proteins | disease resistance protein (TIR-NBS class , putative) |
| Na_454_19854 | 2.13 | 20.1.7.6.1 | at1g73325 | stress.biotic.PR-proteins.proteinase inhibitors | trypsin and protease inhibitor family protein / Kunitz family protein |
| Na_454_40969 | 5.09 | 23.2 | at5g18860 | nucleotide metabolism.degradation | inosine-uridine preferring nucleoside hydrolase family protein |
| Na_454_12178 | 2.35 | 25.5 | at2g38660 | C1-metabolism | tetrahydrofolate dehydrogenase/cyclohydrolase |
| Na_454_23010 | 2.49 | 26.2 | at4g38040 | misc.UDP glucosyl and glucoronyl transferases | exostosin family protein |
| Na_454_35151 | 2.48 | 27.3.11 | at5g22890 | RNA.regulation of transcription.C2H2 | zinc finger (C2H2 type family protein) |
| Na_454_36990 | 2.47 | 27.3.11 | at1g10480 | RNA.regulation of transcription.C2H2 zinc finger family | ZFP5 (ZINC FINGER PROTEIN 5 |
| Na_454_13204 | 2.24 | 27.3.14 | at3g05690 | RNA.regulation of transcription.CCAAT box binding factor | NF-YA2 \| NF-YA2 (NUCLEAR FACTOR Y, SUBUNIT A2 ; transcription factor) |
| Na_454_34042 | 4.27 | 27.3.25 | at1g69560 | RNA.regulation of transcription.MYB domain | MYB105 (myb domain protein 105 |
| Na_454_39997 | 3.28 | 27.3.25 | at1g69560 | RNA.regulation of transcription.MYB domain | AtMYB105 (myb domain protein 105; DNA binding / transcription factor) |
| Na_454_28012 | 2.63 | 27.3.32 | at1g80840 | RNA.regulation of transcription.WRKY domain | WRKY40; transcription factor |
| Na_454_37824 | 4.03 | 27.3.32 | at1g80840 | RNA.regulation of transcription.WRKY domain | WRKY40, ATWRKY40 \| WRKY40; transcription factor |
| Na_454_22020 | 4.04 | 27.3.57 | at5g46910 | RNA.regulation of transcription.JUMONJI family | transcription factor jumonji (jmj) family protein |
| Na_454_32324 | 3.44 | 27.3.62 | at5g08630 | RNA.regulation of transcription.Nucleosome/chromatin | DDT domain-containing protein |
| Na_454_36282 | 2.89 | 28.99 | at3g13610 | DNA.unspecified | oxidoreductase, 2OG-Fe(II oxygenase family protein) |
| Na_454_03136 | 5.81 | 29.4 | at4g24730 | protein.postranslational modification | calcineurin-like phosphoesterase family protein |
| Na_454_00400 | 4.18 | 29.4 | at1g32640 | protein.postranslational modification | ATMYC2, RD22BP1, JAI1, JIN1, MYC2, ZBF1 \|transcription factor |
| Na_454_16548 | 3.29 | 29.5 | at5g09640 | protein.degradation | SNG2, SCPL19; serine-type carboxypeptidase/ sinapoyltransferase |
| Na_454_21861 | 2.36 | 29.5.5 | at2g22980 | protein.degradation.serine protease | SCPL13, Serine-type carboxypeptidase |
| Na_454_19373 | 2.67 | 29.5.7 | at2g45040 | protein.degradation.metalloprotease | matrix metalloproteinase |
| Na_454_40155 | 2.55 | 30.11 | at4g15090 | signalling.light | FAR1 (FAR-RED IMPAIRED RESPONSE 1 ; transcription factor) |
| Na_454_41435 | 2.31 | 30.2.17 | at1g70520 | signalling.receptor kinases.DUF 26 | protein kinase family protein |
| Na_454_27831 | 2.11 | 30.3 | at4g20780 | signalling.calcium | calcium-binding protein, putative |
| Na_454_35735 | 2.58 | 33.1 | at4g37070 | development.storage proteins | PLP1, PLA IVA \| patatin, putative |
| Na_454_14791 | 3.64 | 33.1 | at3g63200 | development.storage proteins | PLP9, PLA IIIB \| PLP9 (PATATIN-LIKE PROTEIN 9 |
| Na_454_33785 | 5.05 | 33.99 | at5g16750 | development.unspecified | TOZ (TORMOZEMBRYO DEFECTIVE |
| Na_454_41066 | 2.12 | 34.12 | at1g05300 | transport.metal | ZIP5; cation transmembrane transporter |
| Na_454_30201 | 2.39 | 34.2 | at3g19930 | transporter.sugars | ATSTP4 \| STP4 (SUGAR TRANSPORTER 4 |
| Na_454_31068 | 2.71 | 34.9 | at1g12480 | transport.metabolite transporters | OZS1, SLAC1, RCD3, CDI3 \| OZS1 (OZONE-SENSITIVE 1 ; transporter) |
| Na_454_26329 | 3.86 | 35.1 | at1g07850 | not assigned.no ontology | transferase, transferring glycosyl groups |
| Na_454_14501 | 3.59 | 35.2 | at5g24740 | not assigned.unknown | protein localization |

b. List of up-regulated genes in MYC2-VIGS plants

| **Probe ID** | **Fold change** | **BIN CODE** | **TAIR no** | **Name** | **Description** |
| --- | --- | --- | --- | --- | --- |
| Na_454_36276 | 5.42 | 34.16 | at1g17840 | ABC transporters and multidrug resistance systems | WBC11 (WHITE-BROWN HOMOLOG COMPLEX PROTEIN 11) |
