# Supplementary material for: NaMYC2 transcription factor regulates a subset of plant defense responses in Nicotiana attenuata
Source: BMC Plant Biol. 2013 May 1;13:73. doi: 10.1186/1471-2229-13-73 (PMC3655906; doi:10.1186/1471-2229-13-73)
Supplement: Additional file 4: Table S3 — List of primers used for the experiments. [file 1471-2229-13-73-S4.docx]

Table S3. **List of primers used for the experiments**

| **Primer** | **FWD sequence (5’->3’)** | **RVS sequence (5’->3’)** |
| --- | --- | --- |
| MYC2_3end-1 | CAACAAGGGATCAAACTATACCG | TTTCCATTTACTGTATTTCTCTTCA |
| MYC2L | CAAGATGTCTAGCCATACT | CCTACAAATTGAGATCTC |
| NaPAL | TGCATACGCTGATGAC | TGGAAGATAGAGCTGTTCGC |
| NaAT1 | TCACAAGGTTCACTTGTGGCTCTG | GCATTTGCCTTGAGTTTGCCTAGG |
| NaMYB8 | AACCTCAAGAAACTCAGGACATACAA | GATGAATGTGTGACCAAATTTTCC |
| NaCV86 | ATCAAATAGCTGAAGATGTC | CCAACAAAGTAGTGCTGTACT |
| NaDH29 | GGCGGGCATTAATTCGTGCTTC | CCAAAAATGATTTGCAAGGTC |
